# The acceleration effect of pump as turbine system during starting period

**DOI:** 10.1038/s41598-023-31899-9

**Published:** 2023-03-25

**Authors:** Yu-Liang Zhang, Jin-Fu Li, Zu-Chao Zhu

**Affiliations:** 1 College of Mechanical Engineering and Key Laboratory of Air-Driven Equipment Technology of Zhejiang Province, Quzhou University, Quzhou, 324000 China; 2 College of Mechanical Engineering, Zhejiang University of Technology, Hangzhou, 310023 China; 3 The Zhejiang Provincial Key Lab of Fluid Transmission Technology, Zhejiang Sci-Tech University, Hangzhou, 310018 China

**Keywords:** Energy science and technology, Engineering, Mathematics and computing

## Abstract

In order to reveal the influence of starting acceleration on starting process of a pump as turbine system, this paper carries out a numerical calculation of the three-dimensional viscous unsteady flow of pump as turbine circulating piping system under three starting acceleration conditions, and obtains the external and internal flow characteristics of each overflow component during the starting process, and also analyzes the energy loss of each component in the piping system in depth with the help of entropy production method and Q criterion method. The results show that during system start-up, the flow rate and outlet static pressure curves of the pump as turbine are hysteresis relative to the rotational speed, the head curve is similar to a linear rise during slow and medium speed start-up, while it shows a parabolic rise during rapid start-up, the entropy production and vorticity in the impeller domain of the pump as turbine are mainly distributed between the blades, and the distribution decreases during start-up. In addition, the pump similarity law does not apply to the performance prediction during the transient start of the pump as turbine.

## Introduction

In recent years, with the increasing energy demand, countries around the world are paying more and more attention to the development and utilization of secondary energy. Centrifugal pump reversal for turbine (referred to as pump as turbine) is widely used in the petrochemical industry for waste liquid residual pressure energy recovery of various devices because of its simple structure, low price, easy installation and maintenance, etc. In normal operation, pumps as turbines often have problems such as unstable operation and narrow efficiency zone. During the start-up process, due to the continuously variable speed operation, the performance parameters such as flow rate, pressure and power will change drastically in a short period, and the internal flow is in an extremely unstable transient flow state, which will easily cause huge pressure pulsation and shock, and then lead to damage to the pump as turbine equipment itself and its connected load equipment^1^. Therefore, it is necessary to conduct a systematic and in-depth study of the transient characteristics of the pump as turbine during the start-up process.

From the published literature, most of the studies have been carried out for steady-state conditions, of which the optimum condition is one of them. Rossi et al.^2^ successfully predicted the optimum condition point performance of pump as turbine using an artificial neural network method. Liu et al.^3^ proposed an iterative flow-based method for predicting the optimum condition point (BEP) under turbine conditions, and the results showed that the developed theoretical model for predicting the performance of pump and turbine conditions was reliable and accurate. Štefan et al.^4^ found that the optimal operating point (BEP) flow and head in turbine conditions are higher than the performance in pump conditions. Miao et al.^5^ proposed a pump as turbine impeller radial surface optimization design method, and the efficiency of the optimized pump as turbine was increased by 2.28% at the optimal service point. Wang et al.^6^ derived a prediction equation for the performance of turbine efficiency point based on pump and turbine efficiency with turbine inlet slip by analyzing the inlet and outlet velocity triangle of the impeller and compared six pumps as turbine with revolutions of 9.0–54.8 for experimental and numerical simulations, and the results showed that the slip coefficient of the pump condition is greater than that of the turbine condition at the design condition point. Frosina et al.^7^ proposed a new method for predicting the performance of centrifugal pumps as hydraulic turbines, which was found to have high accuracy when compared with other methods. Huang et al.^8^ proposed a new theoretical method to predict the flow and head of the pump and turbine at the optimal operating point based on the principle of matching the characteristics between the impeller and the volute. Compared with other prediction methods, the prediction results of the proposed new method were found to be more accurate.

Maleki et al.^9^ numerically calculated the flow characteristics of two fluids with different viscosities within a single-stage and two-stage PAT. The results showed that an increase in viscosity in a single-stage PAT would lead to a decrease in efficiency and an increase in the optimum operating point flow rate; similarly, an increase in viscosity in a two-stage PAT reduced the optimum operating point (BEP) efficiency by 12.5%. Abazariyan et al.^10^ investigated the effect of viscosity on the performance of pump as turbine and found that the reduction in mechanical losses led to higher efficiency when the flow lubrication effect dominated at high viscosities and accordingly proposed a relationship between calculated efficiency and the flow coefficient and Reynolds number. Li^11^ found that the Reynolds number-based flow conversion relational equation is more accurate for predicting the performance of the high-efficiency point in the case of viscosity change, but the accuracy of head prediction still needs further improvement. Zhang et al.^12^ experimentally investigated the transient characteristics of a centrifugal pump reversing as a turbine during an atypical start-up at three steady-state rotational speeds and three steady-state flows. It is found that there is a shocking phenomenon in the rising curve of flow rate and outlet static pressure, and the outlet static pressure shock phenomenon shows a delayed trend with the increase of stable running speed after starting. Li^13,14^ first proposed a method to extract the hydraulic, volumetric, and mechanical efficiency of vortex pumps. The results show that the cavitation performance of the vortex pump as turbine is poor, and its performance conversion coefficients of flow and head are much higher than those of centrifugal pumps with the same speed, and the efficiency of the vortex becomes low as the viscosity increases or the impeller Reynolds number decreases. Hu et al.^15^ studied the hydraulic characteristics of pump as turbine under instantaneous flow conditions. The results show that the efficiency of the pump as a turbine is greatly influenced by the instantaneous flow conditions. As the flow rate increases, the hydrodynamic force on the impeller as well as the pressure fluctuation in the worm gear first decreases and then increases, reaching a minimum value near the design flow rate.

In summary, the current research on pump as turbine is mainly focused on the performance conversion and prediction under steady-state conditions, but the transient characteristics of the pump as turbine start-up process have not yet been studied. Based on this, this paper establishes a circulating piping system including pump as turbine, booster pump, valve and tank, etc., and carries out numerical calculations on the whole circulating piping system to obtain the transient flow characteristics of the pump as turbine, valve, tank and other components. In addition, the transient start-up characteristics of each overflow component, especially the pump as turbine, are further revealed with the help of the dimensionless analysis method, vortex identification method, and entropy production theory.

## Computational models and methods

### Computational models and grid division

**Figure 1:**
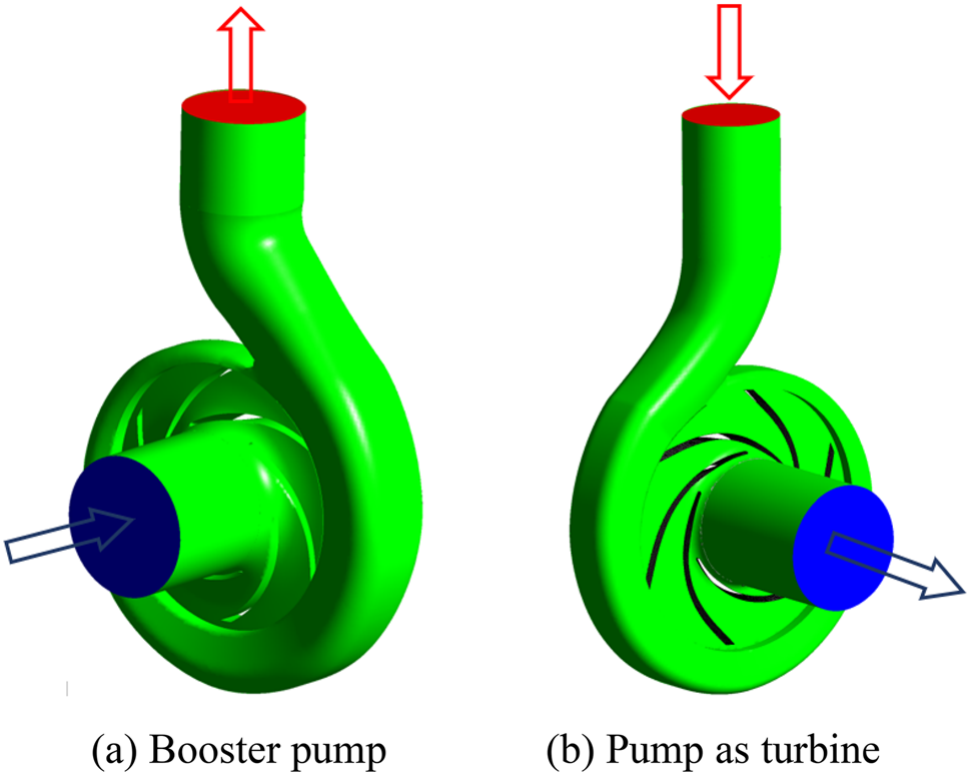
Three-dimensional model. (**a**) Booster pump, (**b**) Pump as turbine.

In this paper, the model of the booster pump is M129-50, whose rated parameters are: *Q*_D_ = 50 m^3^/h, *H*_D_ = 20.54 m, and *n*_D_ = 2900 r/min. The model of the pump as turbine is MH90-25, whose rated parameters are: *Q*_d_ = 25 m^3^/h, *H*_d_ = 20.9 m, and *n*_d_ = 2900 r/min. The model diagrams of the booster pump and pump as turbine are shown in Fig. 1a,b respectively. The number of vanes of both model pumps is 6, and the remaining geometric parameters are shown in Tables 1 and 2, respectively.

**Table 1:** Main geometric parameters of booster pump.

Parameters	Value
Pump inlet diameter *D*_1_ (mm)	76
Pump outlet diameter *D*_2_ (mm)	65
Impeller outlet width *b*_2_ (mm)	14
Impeller diameter *D*_3_ (mm)	137
Outlet angle *β* (*°*)	30

**Table 2:** Main geometric parameters of pump as turbine.

Parameters	Value
Pump inlet diameter *D*′_1_ (mm)	65
Pump outlet diameter *D*′_2_ (mm)	50
Impeller outlet width *b*′_2_ (mm)	10
Impeller diameter *D*′_3_ (mm)	132
Outlet angle *β*′ (*°*)	32

**Figure 2:**
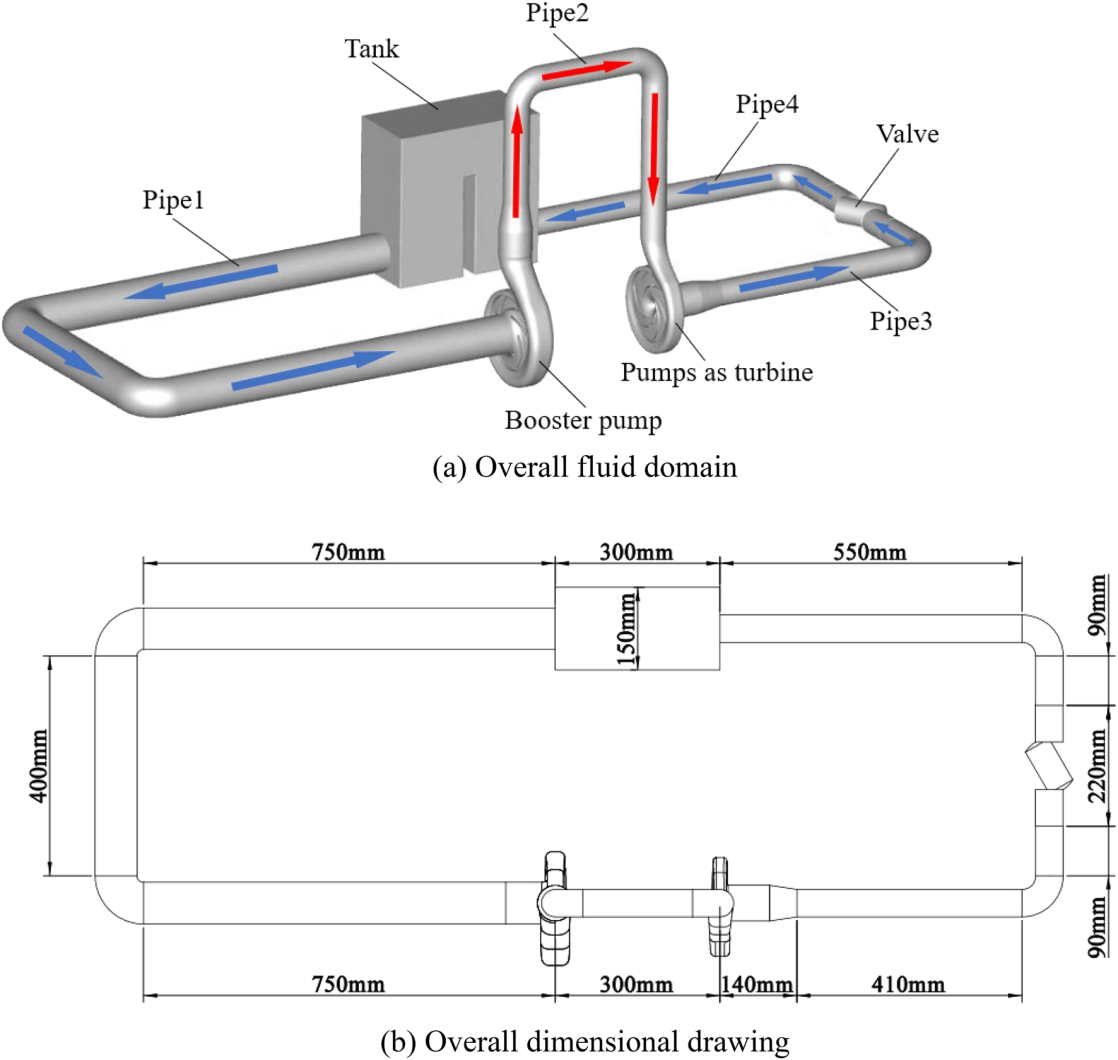
Overall fluid domain of the pump and turbine system. (**a**) Overall fluid domain, (**b**) Overall dimensional drawing.

The circulating piping system constructed in this paper is shown in Fig. 2a. The system is composed of booster pump, pump as turbine, valve, pipe circuit and tank. Among them, in the circulating piping system, the valve is only used to adjust the flow value in the piping system, so it will be simplified to draw. The overall geometry of the circulating piping system is shown in Fig. 2b, the diameter of the outlet pipe of the tank is 76 mm; the inlet diameter of the booster pump is 76 mm and the outlet diameter is 65 mm; the inlet diameter of the pump as turbine is 50 mm and the outlet diameter is 65 mm because the import and export pipe diameters between the pump and turbine are not matched, so the pipe 2 is set as a diffusion pipe at the outlet of the booster pump. In addition, the size of the water tank is 300 × 150 × 300 mm, in which there is a baffle in the middle of the tank, and its size is 25 × 150 × 200 mm. In order to make the simulation results closer to the real situation of the tank, the air pressure on the upper surface of the fluid domain of the water tank is applied to 1 atm in CFD simulation. At a constant speed, the valve opening is adjusted to change the local hydraulic loss and thus the over-flow capacity, i.e., the valve opening can be adjusted to obtain the corresponding stable flow rate and system resistance.

**Figure 3:**
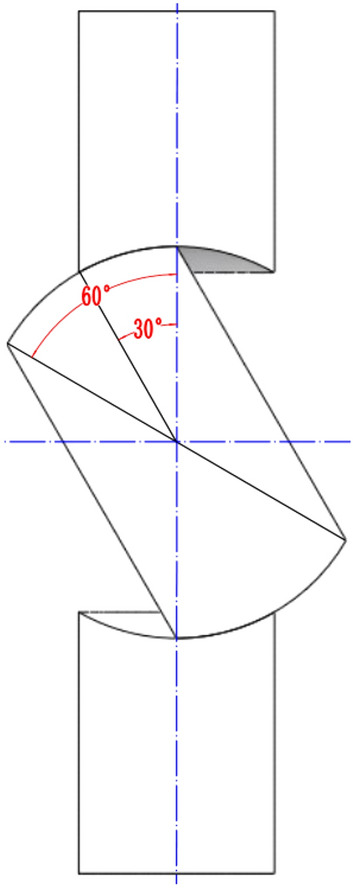
Diagram of valve opening.

The research in this paper is about the effect of the pump as turbine starting acceleration on starting performance. To exclude the influence of flow values in the fluid domain on the pump as turbine starts, the valve opening is set to a constant 0.5. The normal operating steady flow value in the whole fluid domain at this time is about 23.58 m^3^/h. The specific valve opening situation is shown in Fig. 3.

**Figure 4:**
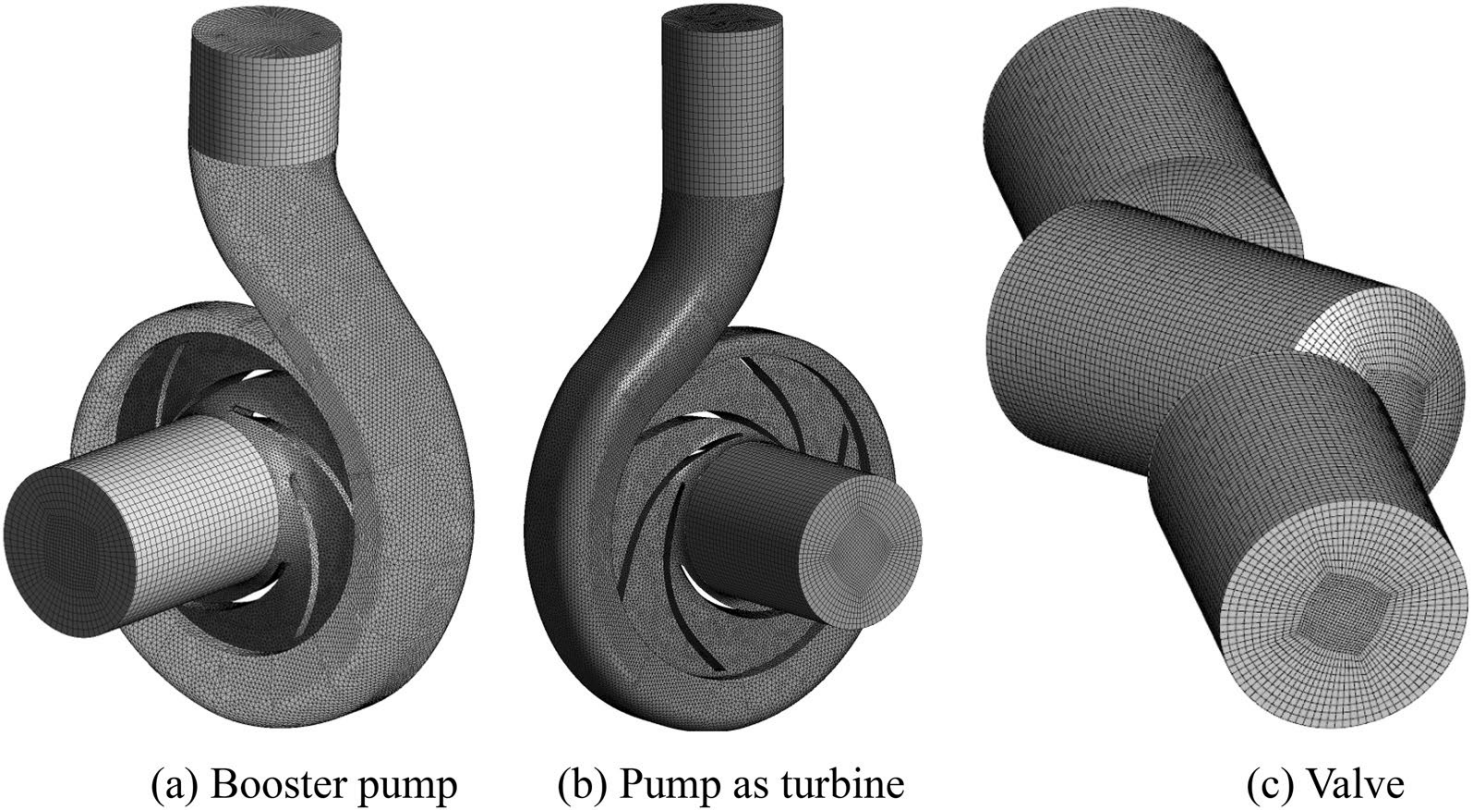
Partial grid diagram. (**a**) Booster pump, (**b**) Pump as turbine, (**c**) Valve.

**Figure 5:**
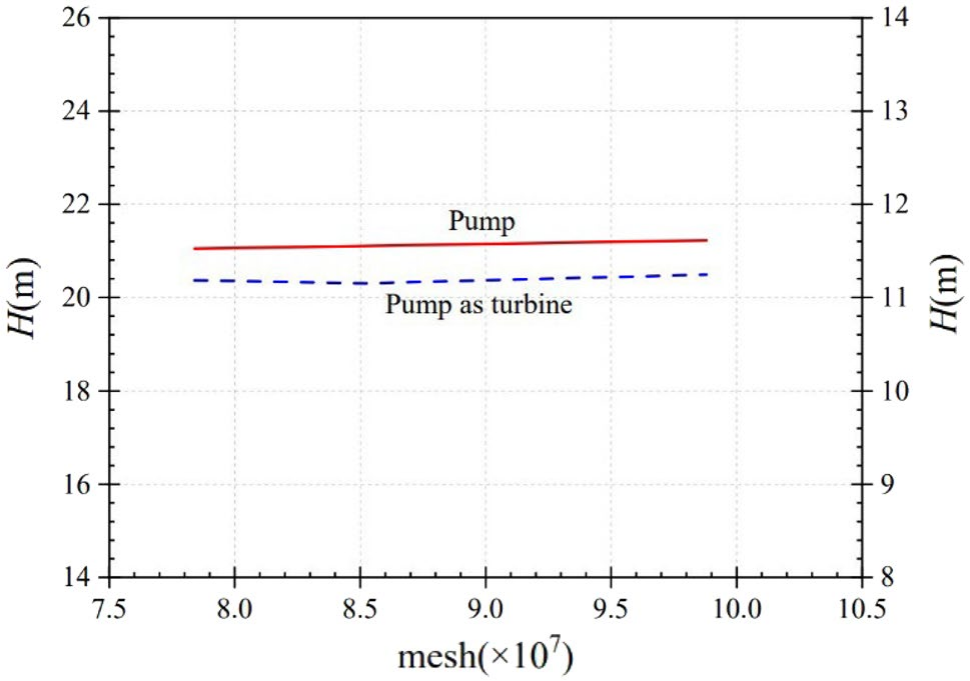
Independence of the grid number.

ICEM CFD 19.2 is used for meshing the overall fluid domain, and the meshes of the booster pump, the pump as a turbine, and the valve are shown in Fig. 4. In order to exclude the influence of the number of grids on the calculation results, the grid independence calculation was performed separately. The effect of the number of grids on the calculation accuracy is shown in Fig. 5. After the grid correlation check, it is found that when the change of calculated head is less than 2%, it is considered that the grid irrelevance requirement is reached. The final mesh number of the whole circulating piping system is 7.84 million after calculation, among which the booster pump and the pump as turbine use tetrahedral non-structural mesh, the numbers are 1.28 million and 1.35 million respectively, and the valve, tank and piping system parts use hexahedral structural mesh, the mesh numbers are 290,000, 3.2 million and 1.72 million respectively. This grid quantity is still slightly insufficient for simulating the micro-fine flow within the boundary layer, but it is sufficient for predicting the external characteristics and capturing the macro-flow structure of the internal flow field. Through the grid quality inspection, the quality of the grid was found to satisfy the requirements.

### Numerical methods

At present, for the computational fluid dynamics calculation of a pump or a pump as turbine during the start-up or shutdown phase, the computational domain is generally a single pump or a pump as a turbine, and then the numerical calculation is performed using a given inlet and outlet boundary conditions. Although the numerical calculation of a single pump or pump as turbine requires a small amount of calculation, in the non-constant calculation because the flow value at the inlet changes with time, and the relationship between the flow value and time need to be obtained in advance through performance experiments. In addition to the above method, the pump, pump as turbine, piping, valves, tanks and other fluid domains can be solved together, although the method is more computational, but does not require prior experiments to obtain the correspondence between the actual flow rate and the change in time.

The basic working process or calculation process of the circulating piping system in this paper is as follows: after starting the booster pump, the parameters such as pressure and flow rate in the fluid domain start to rise, and after the speed of the booster pump reaches stability, the pump is started as a turbine with different starting speeds, and the opening degree of the valve is always kept consistent throughout the starting process, which is 0.5. In order to realize the numerical calculation of different starting speeds of the pump as a turbine, this paper writes In order to realize the numerical calculation of different starting speeds of the pump as turbine, this paper implements the loading of different starting accelerations by writing user-defined functions.

The turbulence model used in this numerical simulation is the RNG *k-ε* model, which is obtained by improving the Standard *k-ε* model^7,16–18^. Compared with the standard *k-ε* model, the RNG *k-ε* model introduces the mainstream time-averaged strain rate in the RNG *k-ε* model to increase the effect of the mean strain rate. the RNG *k-ε* model takes into account the rotational and cyclonic flow conditions in the mean flow, which can better handle the flow with a high strain rate and a large degree of flow curvature. Its form is as follows:

1 $$\rho \frac{{{\text{d}}k}}{{ {\text{d}}t}} = \frac{\partial }{{\partial x_{j} }}\left( {\alpha_{k} \mu_{eff} \frac{\partial k}{{\partial x_{j} }}} \right) + 2\mu_{t} \overline{{S_{ij} }} \frac{{\partial \overline{u}_{i} }}{{\partial x_{j} }} - \rho \varepsilon \,$$

2 $$\rho \frac{{{\text{d}}\varepsilon }}{{{\text{d}}t}} = \frac{\partial }{{\partial x_{j} }}\left( {\alpha_{\varepsilon } \mu_{{{\text{eff}}}} \frac{\partial \varepsilon }{{\partial x_{j} }}} \right) + 2C_{1\varepsilon } \frac{\varepsilon }{k}v_{t} \overline{{S_{ij} }} \frac{{\partial \overline{u}_{i} }}{{\partial x_{j} }} - C_{2\varepsilon } \rho \frac{{\varepsilon^{2} }}{k} - R$$

where $\overline{{S_{ij} }} = \frac{1}{2}\left( {\frac{{\partial \overline{u}_{i} }}{{\partial x_{j} }} + \frac{{\partial \overline{u}_{j} }}{{\partial x_{i} }}} \right)$, $\mu_{eff} = \mu + \mu_{t}$, $\mu_{t} = C_{\mu } \frac{{k^{2} }}{\varepsilon },$ *u* is the speed (m·s^−1^), *ρ* is the density (kg·m^−3^), *k* is the turbulent energy(m^2^·s^−2^), *μ*_eff_ is the effective viscosity coefficient (kg·m^−1^·s), $\overline{{S_{ij} }}$ are the strain rate tensor and *R* is the additional source term in the* ε* equation, representing the effect of the average strain rate *ε*. The expressions are:

3 $$R = \frac{{C_{\mu } \eta^{3} \left( {1 - \eta /\eta_{0} } \right)}}{{1 + \beta \eta^{3} }}\frac{{\varepsilon^{2} }}{k},\quad \eta = Sk/\varepsilon$$

The model parameters in the above equation are *C*_μ_ = 0.0845, *C*_1ε_ = 1.42, *C*_2ε_ = 1.68, *α*_k_ = 1.0, *α*_*ε*_ = 0.769, *β* = 0.012, *η*_0_ = 4.38.

Considering the vicious reason, the no-slip boundary condition is used at the wall, and the coupling of velocity and pressure is realized by the SIMPLEC algorithm. The default under-relaxation factor is used for all variables in the iterative calculation, the time step is set to 0.001 s, and the whole start-up time is 1.5 s. The maximum number of iterations is set to 200 in each time step to ensure absolute convergence in each time step, and the convergence residual is set to 0.001.

### Reliability of numerical method

**Figure 6:**
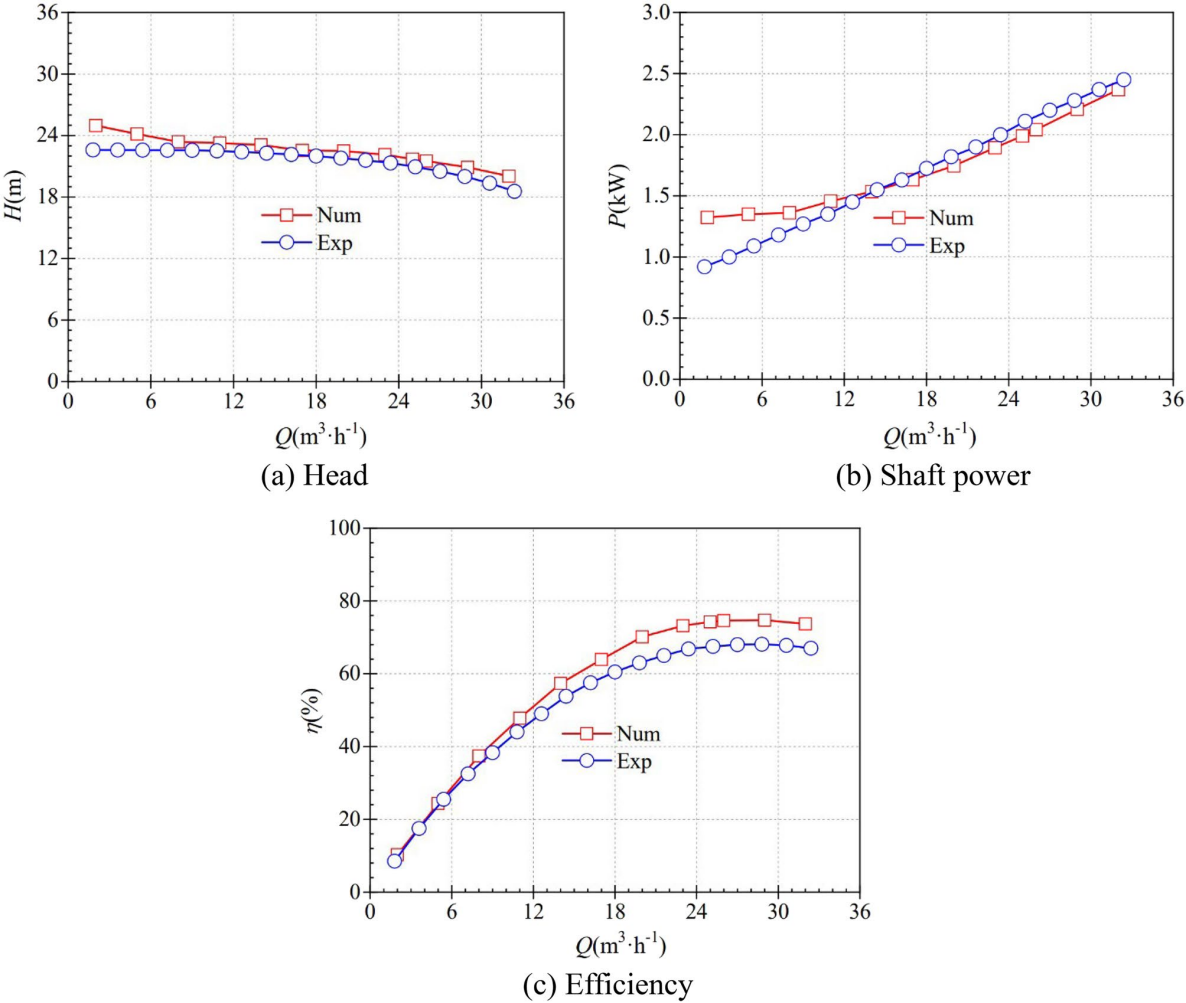
Comparison of external characteristics. (**a**) Head, (**b**) Shaft power, (**c**) Efficiency.

In order to verify the reliability of the numerical calculation method in this paper, the external characteristics of the pump as a turbine model (MH90-25) under pump conditions were first predicted numerically and compared with the experimental results, as shown in Fig. 6. In order to improve the prediction accuracy, the mechanical and volumetric losses are considered in the numerical prediction of the external characteristics under the pump working condition.

Actual pump head

4 $$H = \frac{{\overline{p}_{out} - \overline{p}_{in} }}{\rho g} + \Delta h$$

where $\overline{p}_{out} ,\;\overline{p}_{in}$ are the average total pressure at the pump volute outlet and pump inlet respectively. When considering gravity, Δ*h* is the vertical distance from the pump outlet plane to the center axis of the inlet pipe, and *g* is the acceleration of gravity.

Hydraulic efficiency

5 $$\eta_{h} = \frac{\rho gQH}{{M\omega }}$$

where *Q* is the flow rate, *M* is the impeller torque and *ω* is the angular velocity.

Volumetric efficiency^19^

6 $$\eta_{v} = \frac{1}{{1 + 0.68n_{s}^{{{{ - 2} \mathord{\left/ {\vphantom {{ - 2} 3}} \right. \kern-0pt} 3}}} }}$$

Specific speed

7 $$n_{s} = \frac{3.65n\sqrt Q }{{H^{0.75} }}$$

Each parameter in the formula is taken from the rated working condition value.

Total efficiency

8 $$\eta = \left( {\frac{1}{{\eta_{{\text{v}}} \eta_{{\text{h}}} }} + \frac{{\Delta P_{{\text{d}}} }}{{P_{{\text{e}}} }} + 0.03} \right)^{ - 1}$$

where *P*_*e*_ is the effective output power, $P_{{\text{e}}} = \rho gQH$; Δ*P*_*d*_ is the disc friction loss, calculated according to the following formula:

$$\begin{aligned} & {\text{Where}}\quad n_{{\text{s}}} > 65\quad \Delta P_{{\text{d}}} = 1.1 \times 75 \times 10^{ - 6} \rho gu_{2}^{3} D_{2}^{2} \\ & {\text{Where}}\quad n_{{\text{s}}} < 65\quad \Delta P_{{\text{d}}} = 0.133 \times 10^{ - 3} \rho {\text{Re}}^{0.134} \omega^{3} (D_{2} /2)^{3} D_{2}^{2} \\ \end{aligned}$$

where $${\text{Re}} = 10^{6} \times \omega ({{D_{2} } \mathord{\left/ {\vphantom {{D_{2} } 2}} \right. \kern-0pt} 2})^{2}$$

Shaft power

9 $$P = \frac{\rho gQH}{\eta }$$

At the rated volume flow rate of 25 m^3^/h, the test head, efficiency and shaft power are 21.71 m, 67.50% and 2.11 kW, respectively, and the numerically predicted head, efficiency and shaft power are 20.95 m, 74.23% and 1.99 kW, respectively, so the relative errors are 3.5%, 9.1% and 5.7%, respectively, and each relative error is less than 10%, and the deviations are all within the reasonable. In the whole volume flow range, the predicted head is slightly higher than the test value, but the difference decreases as the volume flow increases and reaches the minimum value near the rated volume flow, after which the difference increases slightly again. In the smaller volume flow range, the predicted power is higher than the test value, but the difference decreases rapidly with increasing volume flow and converges at 17 m^3^/h, after which the test value behaves slightly higher than the predicted value. In the whole volume flow range, the predicted efficiency is slightly higher than the test value, and although the difference increases with the volume flow value, the relative error value is still within a reasonable range. Therefore, considering the mechanical and volumetric losses, the prediction accuracy of the external characteristics is high, which ensures the reliability and accuracy of the mathematical model and the numerical calculation method.

### Calculated solutions

In order to study the influence of the starting acceleration on the starting process of the pump as turbine, different starting conditions of the pump as turbine are defined in this paper, with acceleration times of 0.1 s, 0.6 s and 1.1 s, which are defined as rapid, medium and slow start, respectively, at which the corresponding accelerations are 241.67 r·s^−2^, 40.283 r·s^−2^ and 21.969 r·s^−2^, respectively. In order to exclude the effect of stabilizing speed, the stabilizing speed was kept at the same value after the completion of starting for the three acceleration conditions. The acceleration processes for the rapid, medium and slow start cases are shown by the following equations, respectively:

10 $$n = \left\{ {\begin{array}{*{20}l} {0 \, } & {\quad t < 0.3} \\ {14,500\;(t - 0.3)} & {\quad 0.3 \le t \le 0.4} \\ {1450} & {\quad 0.4 \le t \le 1.5} \\ \end{array} } \right.$$

11 $$n = \left\{ {\begin{array}{*{20}l} 0 & {\quad t < 0.3} \\ {2417\;(t - 0.3)} & {\quad 0.3 \le t \le 0.9} \\ {1450} & {\quad 0.9 \le t \le 1.5} \\ \end{array} } \right.$$

12 $$n = \left\{ {\begin{array}{*{20}l} 0 & {\quad t < 0.3} \\ {1318\;(t - 0.3) \, } & {\quad 0.3 \le t \le 1.4} \\ {1450} & {\quad 1.4 \le t \le 1.5} \\ \end{array} } \right.$$

where *n* is the rotational speed at a given moment, r/min.* t* is the calculation time process, s.

## Results analysis

### Pump as turbine

Pump as turbine circulation piping system specific start-up process is described below: booster pump speed is always maintained at 2900 r/min operation; before 0.30 s, the pump as turbine has not yet started and remains stationary, that is, before 0.30 s, the entire circulation system is in a constant flow state; from 0.30 s onwards, the pump as turbine starts to operate, that is, the speed continues to rise to a stable value. In the pump as turbine speed rising process, the impeller speed is accelerated linearly with three accelerations respectively. The time required for the speed to rise to a stable value is 0.10 s, 0.60 s and 1.10 s respectively, i.e. the start-up process is completed at 0.40 s, 0.90 s and 1.40 s, which are defined as rapid, medium and slow start-up respectively.

**Figure 7:**
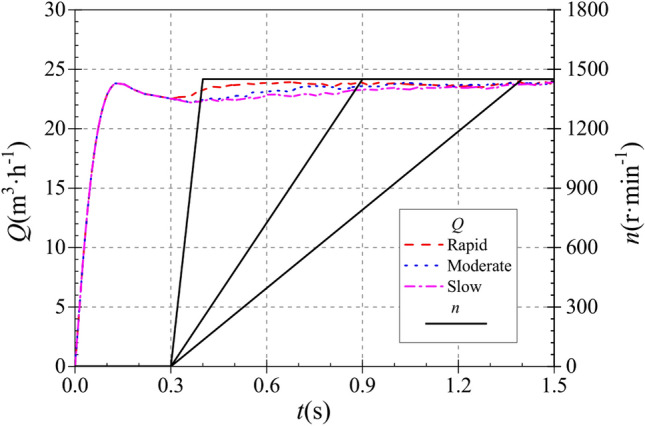
Instantaneous flow rate rise characteristics.

In the entire start-up process, the valve opening is maintained at 0.5, but due to the different start-up acceleration of the pump as turbine under different start-up conditions, which in turn leads to different flow resistance and hydraulic losses in the start-up process, resulting in small differences in the evolution of the flow up curve during the entire start-up process. Figure 7 shows the instantaneous flow curves of the pump as turbine in the circulating piping system under three different start-up accelerations. After the start-up is completed, the stable flow values under the three different start-up accelerations are 23.806 m^3^/h, 23.807 m^3^/h and 23.665 m^3^/h, respectively. The start-up acceleration has a very small effect on the final stable flow, and the small difference is due to the numerical calculation error. In the calculation process, the three flow curves generally show similar evolutionary characteristics, all of which are characterized by a rapid rise to a great value, then a slow decline, and then a slow rise to a stable flow, i.e., the phenomenon of flow shock prevails in the system calculation process. before 0.3 s, since only the booster pump is running in the whole system, the flow changes under the three starting accelerations of rapid, medium and slow are basically the same, all of which reach the maximum flow at the maximum flow value of 23.798 m^3^/h was reached at 0.137 s, after which the flow rate slowly decreased. At 0.3 s, the turbine starts to rotate, and the instantaneous flow rate in the turbine reaches a minimum value of 22.440 m^3^/h for the rapid, medium and slow start accelerations, after which the three flow curves rise at different rates due to the different start accelerations. The three flow curves reached the stable value at 0.655 s, 1.037 s and 1.446 s respectively, while the turbine speed reached the stable value at 0.4 s, 0.9 s and 1.4 s respectively.

It can be seen that there is a certain lag between the time when the flow rate reaches the stable value and the time when the speed reaches the stable value, i.e. the flow rate rises lags behind the speed.

**Figure 8:**
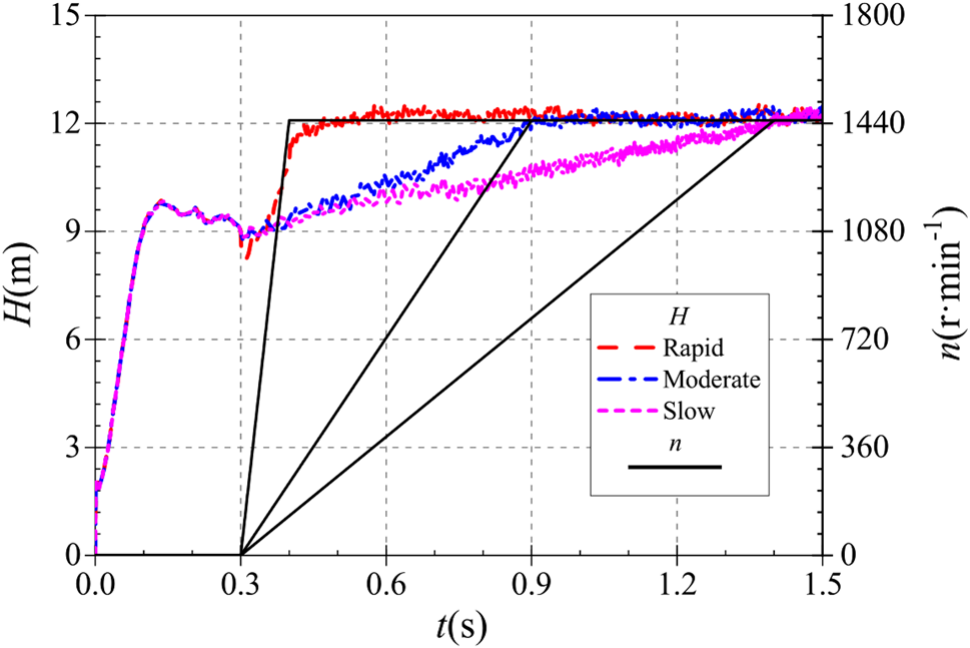
Instantaneous head rise characteristics.

The instantaneous head evolution curves of the pump as turbine under different starting accelerations are shown in Fig. 8. It is obvious to find that the overall trend of head change under different starting accelerations remains the same during the calculation, all of which are fast growing, then slowly decreasing, and then rising to a stable value. Before 0.3 s, because the pump as turbine does not operate under the three operating conditions, its head curve rises in the same way, all rising rapidly first, reaching a local maximum value of 9.86 m at 0.136 s. After reaching a local maximum point, it fluctuates and reaches a local minimum value of 9.02 m at 0.3 s. After the calculation time of 0.3 s, the pump as turbine starts operating at three different starting accelerations. After the calculation time of 0.3 s, the pump as turbine starts to run with three different starting accelerations, and there are some differences in the head rise curves under different starting conditions. The rotation of pump as turbine impeller generates certain pressure loss, and the loss increases gradually as the pump turbine speed rises, so the head rises significantly during the pump turbine start-up process. In the process of a slow start, the head curve of the turbine shows a linear growth trend and is highly correlated with the speed growth law; in the process of medium speed start, the head curve also has a high similarity with the speed growth law, showing a similar linear growth trend. Unlike the slow and medium speed start, the head curve during the rapid start shows a parabolic rise; at the beginning of the start, the instantaneous head first decreases rapidly, then increases rapidly, and finally increases slowly to a stable value. At 0.309 s, the instantaneous head of the pump as turbine reaches a local minimum value of 8.282 m, which is 0.741 m lower compared with the head at 0.3 s. After that, the head curve starts to rise rapidly, and at 0.417 s, the instantaneous head of the pump as the turbine is 11.782 m, and at 0.6 s, the instantaneous head of the pump as the turbine is 12.044 m. It can be seen that in the rapid start. It can be seen that under the rapid start condition, the evolution of the instantaneous head curve of the pump for turbine and the speed growth law are different, and do not show a linear growth law.

In summary, the evolution of the head curve of the pump as turbine remains highly consistent in the three calculation cases before the turbine starts; after the pump as turbine starts, the head curve and the speed curve grow similarly in the slow and medium speed start process, both showing a similar linear growth trend; in the rapid start process, the head curve shows a parabolic growth, and there is a sudden drop in the head at the beginning of the turbine start. During the rapid start, the head curve shows a parabolic growth, and there is a sudden drop in the head at the beginning of the turbine start.

**Figure 9:**
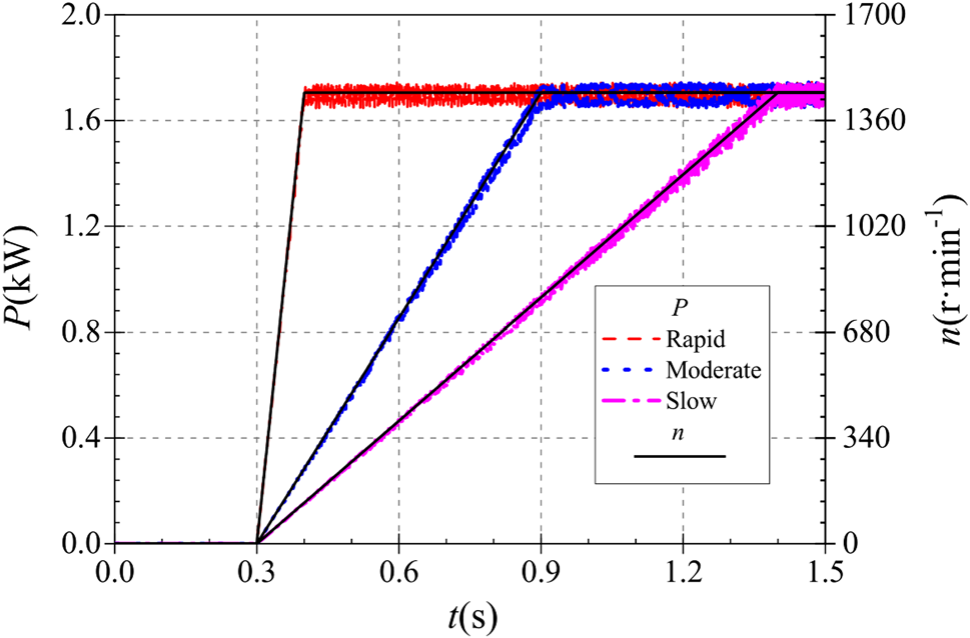
Instantaneous shaft power rise characteristics.

The instantaneous shaft power curves of the pump as turbine under different starting acceleration conditions are shown in Fig. 9. The average values of the steady shaft power at the end of the three different start-up accelerations are 1.698 kW, 1.698 kW and 1.698 kW, respectively. it can be seen that despite the different start-up accelerations, the steady shaft power values are the same since the same steady speed is reached at the end of the start-up. During the starting process, the shaft power curve generally shows a linear increase and starts to show periodic fluctuations after reaching the stable value. At 0.4 s of the calculation process, the rapid-starting pump as turbine reaches a stable value of 1.689 kW; at 0.9 s of the calculation process, the medium-speed starting pump as turbine reaches a stable value of 1.694 kW; at 1.4 s of the calculation process, the slow-starting pump as turbine reaches a stable value of 1.697 kW. It can be found that the pump as turbine impeller speed reaches the stable value at 0.4 s, 0.9 s and 1.4 s for rapid, medium and slow start accelerations, respectively, while the instantaneous shaft power of the pump as turbine reaches the stable value at the same time as above.

**Figure 10:**
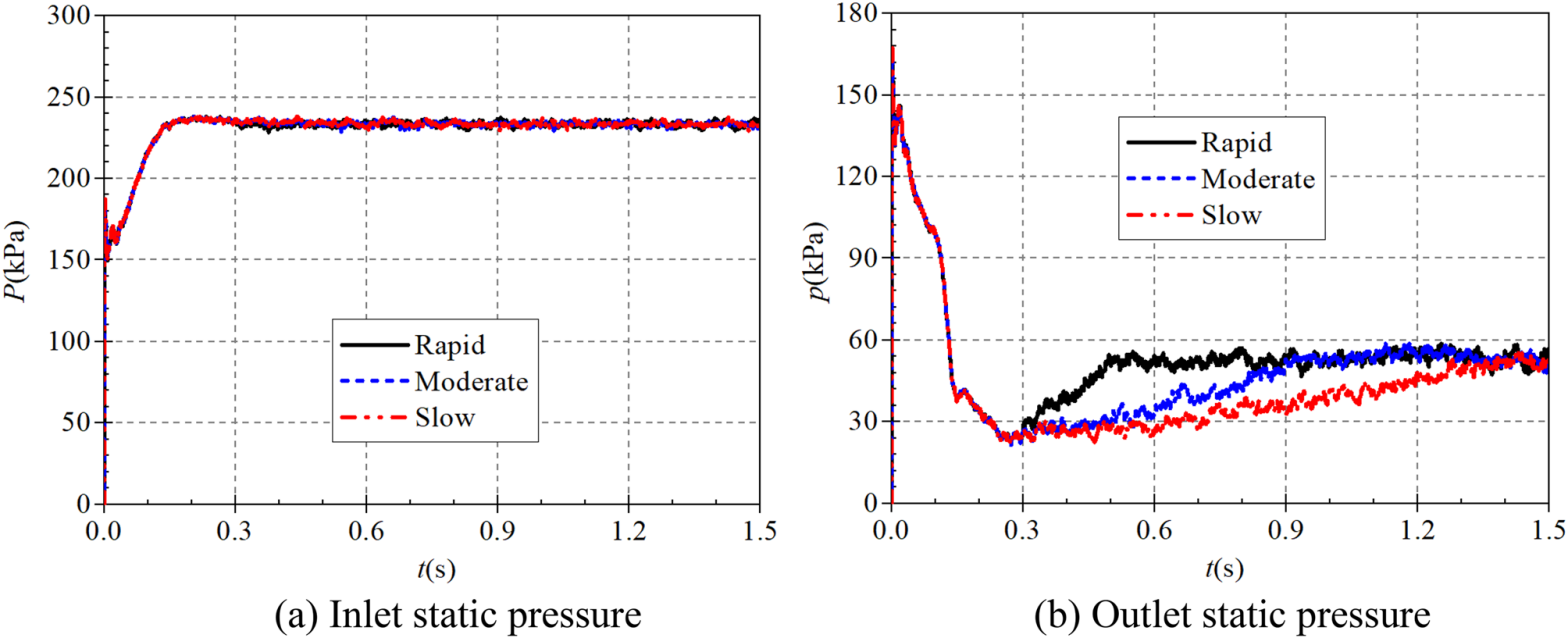
Instantaneous static pressure rise characteristics. (**a**) Inlet static pressure, (**b**) Outlet static pressure.

In the rapid, medium and slow starting conditions, the instantaneous static pressure curves at the pump as turbine inlet and outlet are shown in Fig. 10. Figure 10a shows the instantaneous static pressure at the turbine inlet. It can be found that the instantaneous static pressure curves at the pump as turbine inlet under different starting acceleration basically overlap, which is because the pressure at the turbine inlet is determined by the pressure at the outlet of the booster pump, so the effect of the rotation of the pump as turbine impeller on its inlet pressure is almost non-existent. At the beginning of the calculation process, the pressure at the inlet of the pump as turbine showed a general trend of fluctuating up and down first, then rising rapidly and finally falling slowly to a stable value. The pressure at the pump as turbine inlet fluctuates sharply until 0.03 s, then reaches a local extreme value of 237.861 kPa at 0.209 s, and finally reaches a stable value of 233.259 kPa at 0.31 s. It can be seen that the pump as turbine inlet pressure is not related to its impeller rotation speed, and there is a slight pressure shock phenomenon during the start-up process.

Figure 10b shows the instantaneous static pressure curves at the outlet of the pump as turbine, unlike the inlet static pressure, the outlet static pressure shows a relatively complex trend, and all three curves show a trend of first falling and then rising to a stable value. In the calculation process of 0.017 s, 0.092 s and 0.167 s, there are obvious extreme value points, the value of 146.646 kPa, 101.581 kPa and 41.926 kPa respectively. 0.3 s after the calculation process, the pump turbine starts to start, and the impeller speed rises continuously. Due to the different starting accelerations of the pump as turbine impeller, the three outlet static pressure curves show different rising characteristics. In the rapid start-up process, the pump as turbine outlet static pressure reached a stable value of 52.983 kPa at 0.524 s. Before that, the pump as turbine outlet static pressure showed a fluctuating rising trend; the time required for the outlet static pressure to rise to the stable value showed a slight tendency to extend compared with the rising impeller speed of the pump as turbine. During the medium speed start-up, the pump as turbine outlet static pressure reaches a stable value of 53.321 kPa at 0.979 s. During the slow speed start-up, the pump as turbine outlet static pressure reaches a stable value of 55.391 kPa at 1.444 s. Thus, it can be seen that the time required for the pump as turbine outlet static pressure to reach a stable value has a slight hysteresis concerning the speed curve; and with the increase of the start-up acceleration, the outlet static pressure also has a slight hysteresis. The outlet stabilized static pressure also decreases slightly with the increase of starting acceleration.

**Figure 11:**
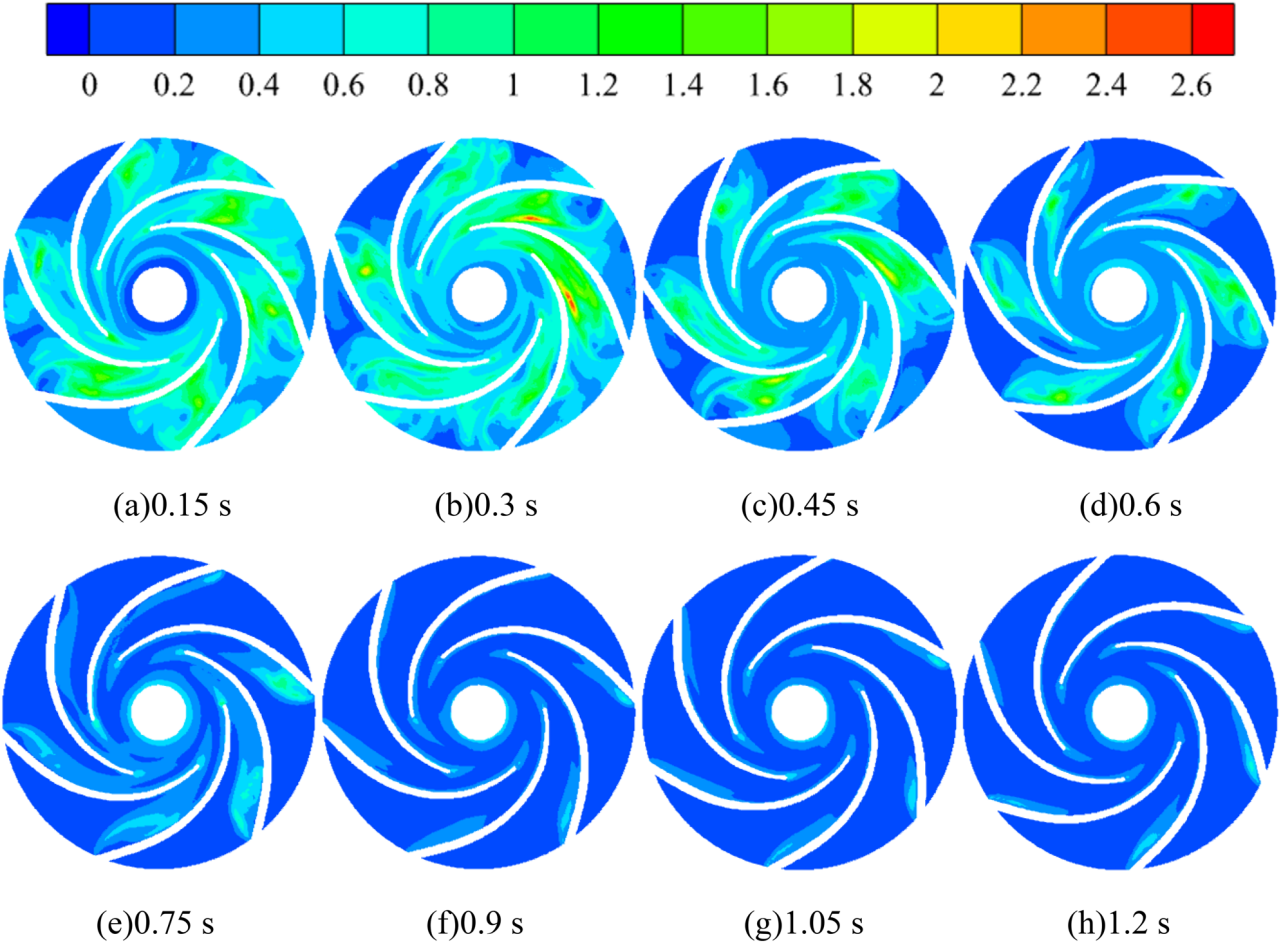
Turbulent kinetic energy distribution of impeller runner section during medium-speed start-up (m^2^/s^2^).

Turbulent kinetic energy is a measure of the development or dissipation of turbulence, and its size and the inhomogeneity of the distribution reflect the pulsation diffusion range and the size of viscous dissipation losses, the larger the turbulent kinetic energy, the more active the small-sized flow structure in the turbulent flow. Figure 11 shows the turbulent kinetic energy distribution of the turbulent impeller cross-section at different starting moments of the pump for turbine impeller during medium-speed start-up. During the whole calculation process, the turbulent kinetic energy in the impeller runner shows a trend of first increasing and then decreasing before stabilizing. The turbulent kinetic energy is maximum at 0.3 s and minimum at 0.9 s. This is because in the calculation process, the pump as turbine impeller is stationary before 0.3 s, when the fluid output from the booster pump directly impacted the stationary vane, which led to great flow loss; after 0.3 s, the pump as turbine impeller began to rotate, when the impact on the vane began to gradually reduce, that is, the turbulent kinetic energy decreased; after 0.9 s, the pump as turbine impeller maintained a uniform rotation, when the turbulent kinetic energy did not change significantly. It is obvious to find that, at the calculation time of 0.3 s, the turbulent kinetic energy distribution area in the flow channel of the pump as turbine impeller is very large, The larger turbulent kinetic energy distribution between any two blades is more obvious, among which the turbulent kinetic energy distribution near the location of the VII section of the worm shell is the most intense, and its maximum value can reach 2.6 m^2^/s^2^. After the calculation time of 0.9 s, the turbulent kinetic energy value in the impeller runner is small and the distribution is also very low, and the turbulent kinetic energy value in the whole impeller runner is reduced to about 0.6 m^2^/s^2^, and the turbulent kinetic energy is mainly concentrated near the blade tip and impeller outlet at this time. In summary, the turbulent kinetic energy distribution in the impeller runner of the pump as turbine decreases with the increase of the pump as turbine impeller speed during the pump as turbine start-up process, and the turbulent kinetic energy distribution in the impeller runner becomes more uniform after the acceleration is completed.

**Figure 12:**
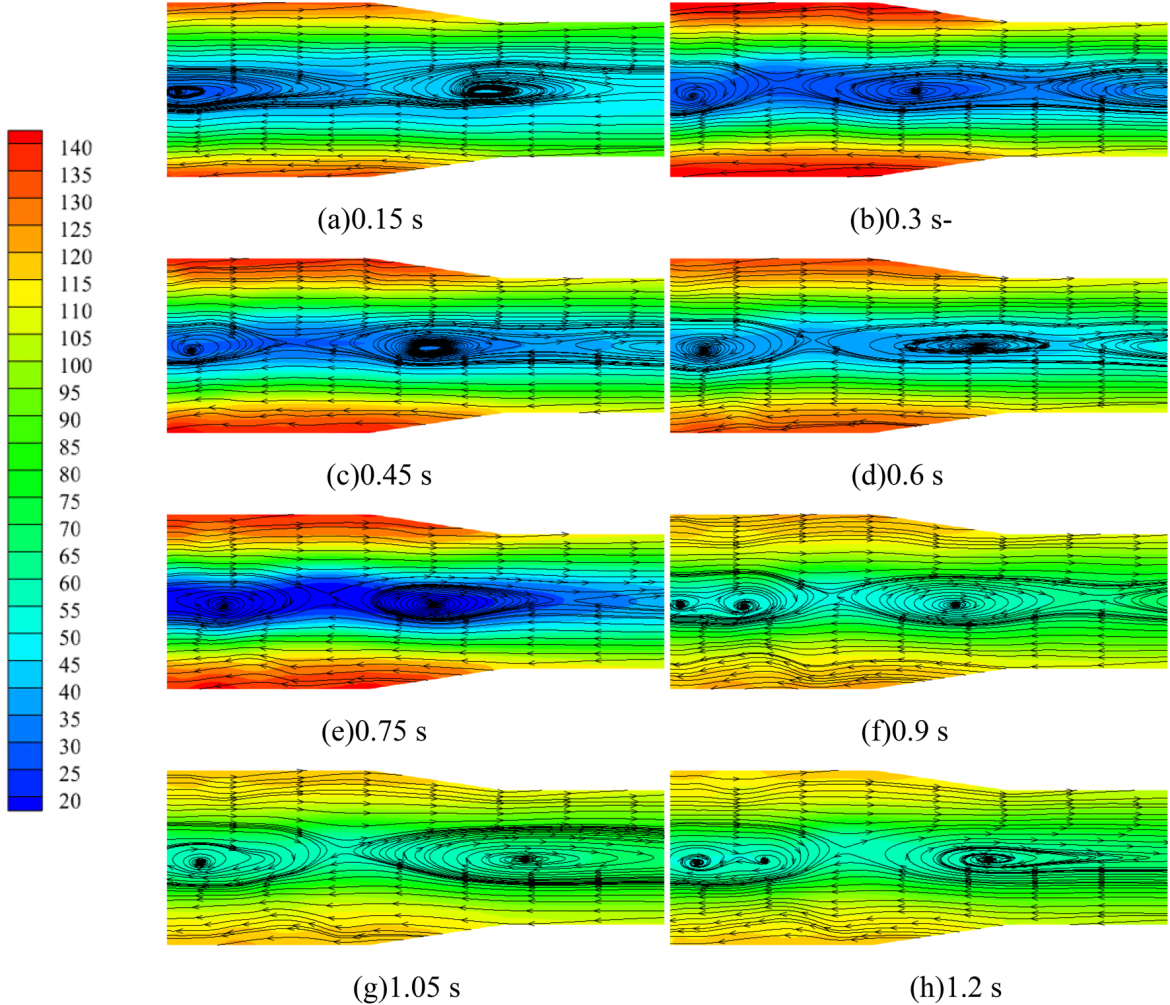
Pressure-flow line diagram of pump as turbine tail pipe during medium-speed start-up (kPa).

Figure 12 shows the pressure distribution and the spatial and temporal evolution of the flow line of the tailpipe at the outlet of the pump as turbine at different moments during the medium-speed start-up. As can be seen from the figure, the entire calculation process of the pump as turbine outlet flow channel, the pressure distribution shows the characteristics of high on both sides and low in the middle, especially in the tailpipe diameter is larger, the maximum pressure and the minimum pressure difference at this location up to 120 kPa; in the back end of the tailpipe location, the pressure difference between the two ends of the pipeline and the middle is further reduced. In the flow distribution, although the outlet flow channel is relatively simple, there are multiple vortices in the outlet flow channel cross-section; in the calculation process of 0.15 s there are two vortex areas distributed in the middle of the longitudinal position of the outlet flow channel, of which the central pressure of the left vortex is about 30 kPa, while the central pressure of the right vortex is relatively large about 50 kPa. With the increase of impeller rotation speed, the vortex position is continuously shifted to the right; with the increase of impeller rotation speed, the overall pressure value of the vortex center position shows a gradually increasing trend, but in 0.75 s the pressure value of the vortex center position is very small, from the left to the right two vortex center pressure value is about 25 kPa and 30 kPa; at any point in time, the pressure value of the vortex center position from left to right gradually increase. In the impeller rotation stability, the number of vortex areas again reduced to two. The reason for the above changes is that the pump as turbine impeller in the process of accelerating the start, the speed is increasing, and the flow through the pump as turbine is increasing. When the passing flow rate is small, the flow separation phenomenon in the tailpipe is serious, the inhibition ability of the mainstream to the boundary layer separation is insufficient, and the vortex area is wide and many areas; when the passing flow rate increases, the flow separation phenomenon in the tailpipe is suppressed, the inhibition ability of the mainstream to the boundary layer separation is obviously enhanced, and the vortex area range is compressed.

**Figure 13:**
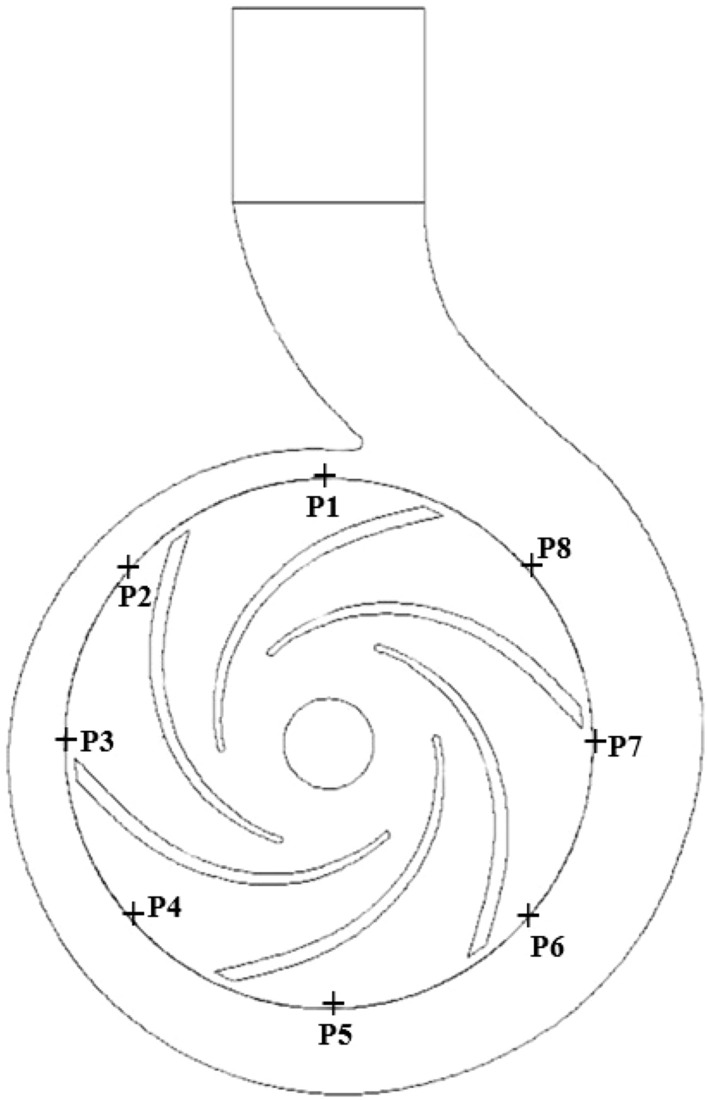
Pressure pulsation monitoring point (pump as turbine).

Due to the dynamic and static interference between the rotating impeller and the stationary volute in the pump as turbine, as well as the combined effect of the rotating stall and wake-jet structure, the internal flow field becomes extremely complex and will show non-constant disturbance flow characteristics. This disturbed flow will cause periodic pressure pulsation in the flow field, and the fluid will transfer the pressure pulsation to the impeller and volute, which will cause the vibration and noise of the pump as turbine. Therefore, the analysis of the pressure pulsation can effectively show the turbulence intensity of the fluid. Based on this, a series of monitoring points are set up in the volute casing of the pump as turbine to monitor the internal pressure pulsation, as shown in Fig. 13. In addition, in this paper, the pressure coefficient is used to dimensionless the transient pressure, and the calculation formula is:

13 $$C_{p} = \frac{{p - \overline{p} }}{{\frac{1}{2}\rho U_{2}^{2} }}$$

where *U*_2_ is the pump as turbine impeller inlet (pump impeller outlet) circumferential velocity, m/s; *p* is the transient static pressure, Pa; $\overline{p}$ is the average static pressure, Pa; *ρ* is through the medium, that is, the density of water, kg/m^3^.

**Figure 14:**
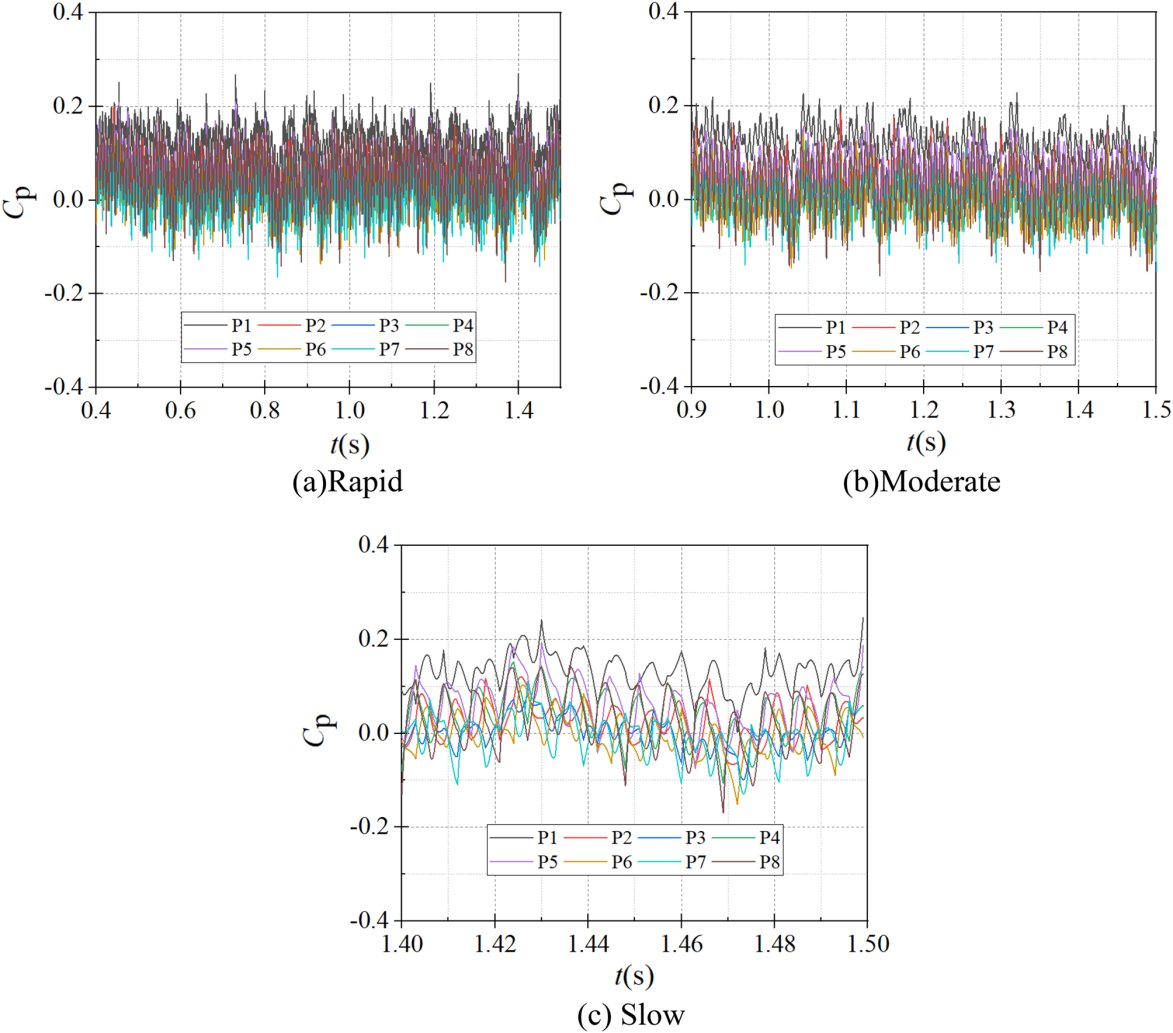
Frequency domain diagrams of different monitoring positions in the volute. (**a**) Rapid, (**b**) Medium, (**c**) Slow.

The time domain diagrams of different monitoring points in the worm shell at the end of the starting process under different starting conditions are shown in Fig. 14. As can be seen from the figure, at the rapid start, the average pressure coefficients at each monitoring point are 0.134, 0.035, 0.023, 0.053, 0.062, 0.012, 0.010 and 0.049; at the medium start, the average pressure coefficients are 0.125, 0.021, 0.008, 0.039, 0.054, 0.0004, − 0.004 and 0.034; at the slow start, the average pressure coefficients were 0.131, 0.024, 0.002, 0.042, 0.061, − 0.001, − 0.007 and 0.029, It is obvious that the pressure coefficient at monitoring point 1 near the volute tongue is the largest; the pressure coefficients at monitoring points P3, P6 and P7 are relatively small, i.e., the pressure coefficients near sections III, VI and VII are small; at the medium and slow start, the pressure coefficient at monitoring point P7 near the VI section of the volute is negative.

At the same time for the instantaneous pressure pulsation, the magnitude of its pulsation can reflect the degree of fluid flow disorder and hydraulic loss at the observed location to a certain extent, and the instantaneous pressure fluctuation magnitude is used to better characterize the fluid flow and hydraulic loss^20^. The fluctuation magnitude is defined as:

14 $$C_{{\text{A}}} = \, \left( {P_{\max } - P_{\min } } \right){ /}P_{\max }$$

The pressure fluctuations at different monitoring points in the volute at the end of the start-up process are shown in Table 3. In different starting situations, due to the rotation of the impeller, the instantaneous pressure at the pressure monitoring points from P1 to P8 shows a sharp fluctuation change. For the same monitoring point, its pressure fluctuation amplitude decreases with the increase of the pump as turbine impeller starting acceleration, and the pressure fluctuation amplitude is the largest at the slow start; for the same start acceleration situation, the pressure fluctuation amplitude is the largest at the monitoring point near the VIII section, and the fluctuation amplitude is the smallest at the monitoring point near the volute.

**Table 3:** Pressure fluctuations at different monitoring points of the volute.

	Pressure fluctuation amplitude							
	P1 (%)	P2 (%)	P3 (%)	P4 (%)	P5 (%)	P6 (%)	P7 (%)	P8 (%)
Rapid start-up	5.963	6.879	5.878	7.219	7.724	6.521	7.321	9.008
Medium start-up	7.106	9.259	9.109	10.872	9.975	11.621	10.848	11.644
Slow start-up	8.474	11.086	11.179	13.845	12.841	12.569	13.008	15.268

**Figure 15:**
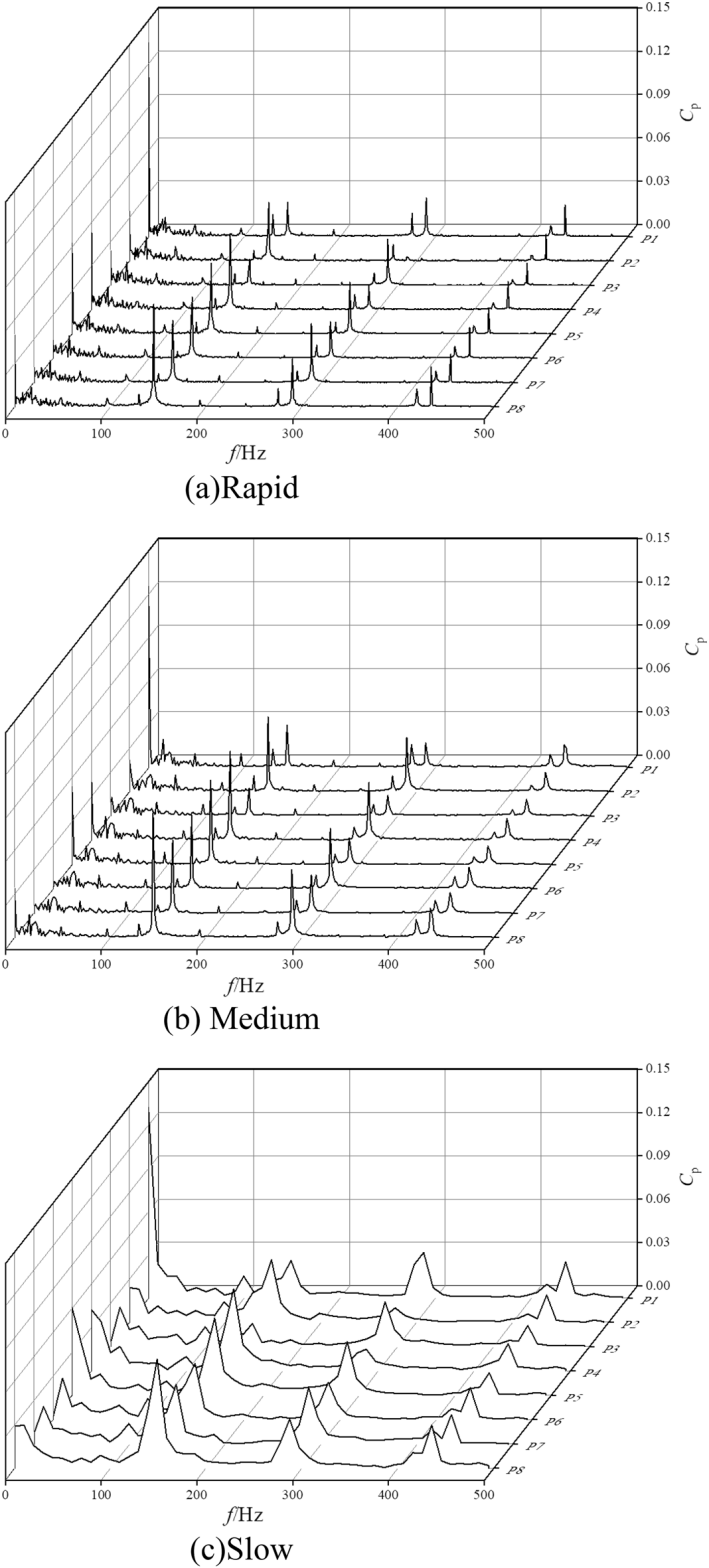
Frequency domain diagram of different monitoring positions in the worm shell. (**a**) Rapid, (**b**) Medium, (**c**) Slow.

At present, in the frequency domain analysis, Fast Fourier Transform is mainly used to get the global frequency characteristics. The expression between the speed *n*_max_ and the shaft frequency *f*_z_ of the pump as turbine during the start-up process is shown in Eq. (15). Figure 15 shows the frequency domain of the pressure pulsation at different monitoring points in the volute channel at the end of the start-up process for different start-up acceleration scenarios. It can be seen that under different start-up acceleration scenarios, the larger values of the pressure pulsation spectrum in the volute channel are mainly concentrated in the low to medium frequency region within 300 Hz. The actual main frequency of each monitoring point is 145.32, 144.75 and 148.51 Hz for the rapid, medium and slow start-up acceleration scenarios, and the impeller frequency is 6*f*_z_ because the number of impellers of the pump as turbine is 6. The difference between the theoretical main frequency and the actual main frequency is not significant. In the rapid start case, the peak principal frequencies of the eight monitoring points are 0.023, 0.041, 0.017, 0.052, 0.048, 0.042, 0.043, 0.071; in the medium speed start case, the peak principal frequencies are 0.029, 0.051, 0.019, 0.061, 0.058, 0.051, 0.050, 0.086; in the slow start case, the peak principal frequencies are 0.029, 0.051, 0.019, 0.061, 0.058, 0.051, 0.050, 0.086. 0.086; in the slow start case, 0.029, 0.043, 0.016, 0.056, 0.053, 0.039, 0.041, 0.075. It can be found that the amplitude is the smallest on section II and the largest on section VIII.

15 $$f_{z} = \frac{{n_{\max } }}{60}$$

where *n*_max_ is the pump as turbine start after the end of stable operation speed, r/min.

The atypical start-up process is described in terms of dimensionless volumetric flow rate, dimensionless head and dimensionless shaft power over time^21^. The three are defined as:

16 $$\left\{ {\begin{array}{*{20}l} {\phi (t) = Q(t)/\pi D_{2} b_{2} u_{2} (t)} \\ {\psi (t) = 2{\text{g}}H(t)/u_{2}^{2} (t)} \\ {\Phi (t) = P(t)/\rho D_{2}^{2} u_{2}^{3} (t)} \\ \end{array} } \right.$$

where *u*_2_(*t*) is the instantaneous circumferential velocity at the impeller outlet and its expression is $u_{2} (t) = \pi D_{2} n(t)/60$.

**Figure 16:**
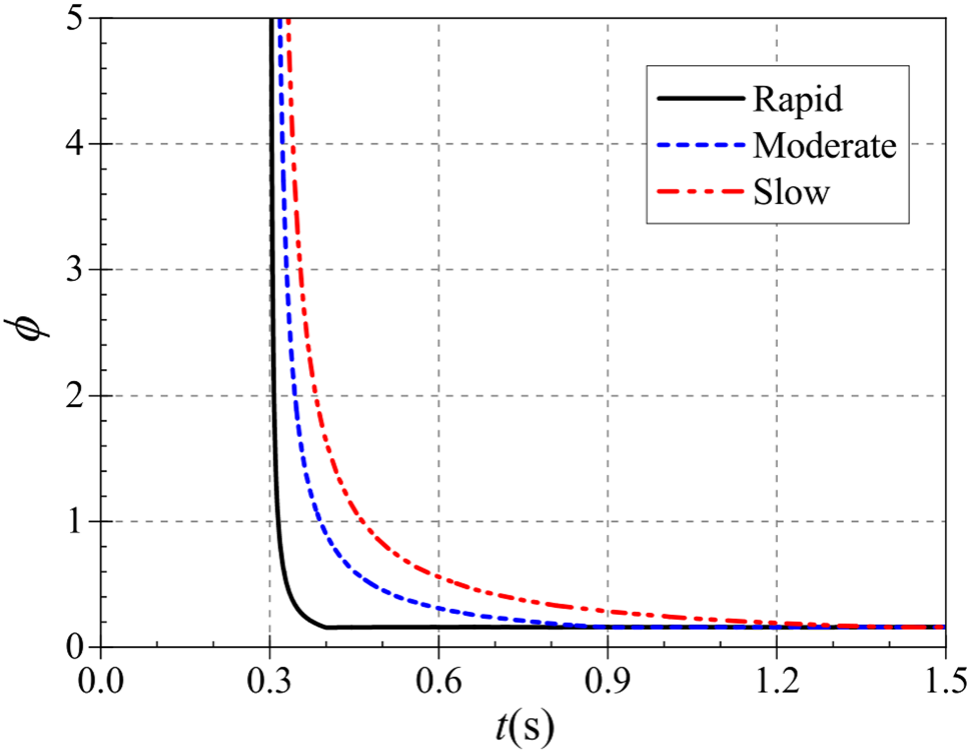
Dimensionless flow coefficient.

Figure 16 shows that the trends of the dimensionless flow coefficients during the start-up of the pump as turbine are generally similar for different start-up accelerations. During the start-up process, the dimensionless flow coefficients were all extremely large at 0.3 s, and the evolution of the flow coefficient curves was characterized by a rapid decrease from the extremely large value first, followed by a slow decrease to the final stable value. However, the time to drop from the very large value to the stable value is different for different start-up accelerations. During the rapid, moderate and the slow start, the dimensionless flow coefficients reach stable values at 0.4 s, 0.9 s and 1.4 s, respectively, and their corresponding stable values are 0.1555, 0.1575 and 0.1586, respectively. Thus, it can be seen that the start acceleration has little effect on the dimensionless flow coefficients, and the time to reach the stable values is highly consistent with the end of the start.

**Figure 17:**
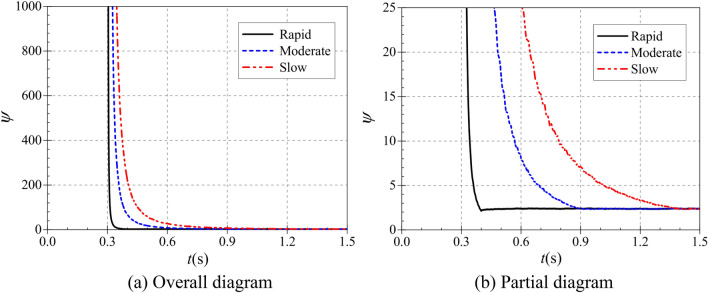
Dimensionless head coefficient. (**a**) Overall diagram, (**b**) Local diagram.

Figure 17 shows the variation of the dimensionless head coefficient during the pump as turbine start-up. Compared with the dimensionless flow coefficient, both have the same trend of rapid decrease from the extreme point and then slowly decrease to the stable value. But in terms of values, the stable head system is much larger, with a difference of nearly 15 times between the two stable values. From the overall diagram, the pump as turbine starts to operate at 0.3 s from the calculation moment, and its dimensionless head also starts to decrease from the maximum value at 0.3 s from the calculation moment. From the local graph, the dimensionless head coefficient reaches its stable value at 0.486 s, 0.900 s and 1.400 s, and its stable values are 2.3834, 2.3496 and 2.3824, respectively. it can be seen that the time to reach the stable value of the dimensionless head coefficient during the start-up process is also related to the acceleration time of the pump as turbine, and there is a slight delay in the rapid start-up.

**Figure 18:**
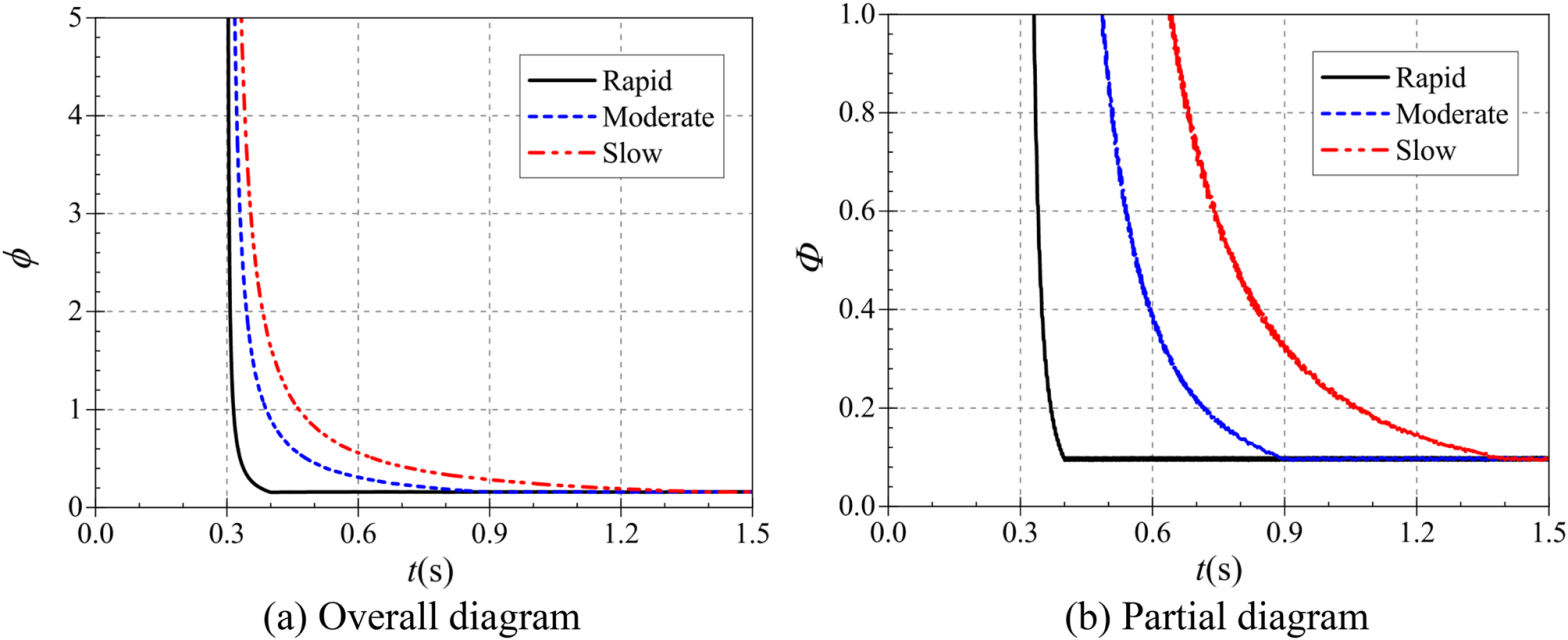
Dimensionless power coefficient. (**a**) Overall diagram, (**b**) Partial diagram.

Figure 18 shows the variation of the dimensionless power coefficient during the start of the pump as turbine. In line with the variation pattern of the dimensionless flow and head coefficients, it still decreases rapidly from the extreme point and then slowly decreases to a stable value. The pump as turbine starts operation from 0.3 s at the calculation time, and the dimensionless head coefficient reaches the stable value at 0.4 s, 0.9 s and 1.4 s at the calculation time, and its stable values are 0.0963, 0.0962 and 0.0971, respectively. it can be seen that the time for the dimensionless power coefficient to reach the stable value during the start-up process is also related to the pump as turbine start-up speed.

The separation region within the pump as turbine is analyzed in depth with the help of the vortex identification method. From the second-order tensor characteristic, the characteristic equation of the local velocity gradient tensor $\nabla V$ of the incompressible flow of the centrifugal pump can be written as:

17 $$\lambda^{3} + Q_{*} \lambda - \det \nabla V = 0$$

If *λ*_1_, *λ*_2_, *λ*_3_ are its 3 roots, there exist 3 independent invariants between them:

18 $$\left\{ \begin{gathered} E_{11} + E_{22} + E_{33} = \lambda_{1} + \lambda_{2} + \lambda_{3} = divV = 0 \hfill \\ Q_{*} = - \lambda_{1} \lambda_{2} - \lambda_{2} \lambda_{3} - \lambda_{3} \lambda_{1} = \frac{1}{2}\left( {\left\| \Omega \right\|^{2} - \left\| E \right\|^{2} } \right) \hfill \\ \det \nabla V = \lambda_{1} \lambda_{2} \lambda_{3} \hfill \\ \end{gathered} \right.$$

where $E_{ij} = \frac{1}{2}\left( {\nabla_{i} V_{j} + \nabla_{j} V_{i} } \right)$ is the strain rate tensor and $\Omega_{ij} = \frac{1}{2}\left( {\nabla_{i} V_{j} - \nabla_{j} V_{i} } \right)$ the vortex tensor $\left\| E \right\|^{2} = \sum\nolimits_{i,j = 1}^{3} {E_{ij}^{2} } ;\quad \left\| \Omega \right\|^{2} = \sum\nolimits_{i,j = 1}^{3} {\Omega_{ij}^{2} }$.

In this paper, the vortex region is analyzed using the *Q* criterion. Hunt et al.^22,23^ proposed to define the region with Q_*_ > 0 as a vortex, which means $\left\| \Omega \right\|^{2} > \left\| E \right\|^{2}$, i.e., the rotation of the fluid (vortex magnitude) plays a dominant role in the region of the centrifugal pump vortex, while the strain rate magnitude of the fluid is secondary, and this approach is called the *Q* criterion.

**Figure 19:**
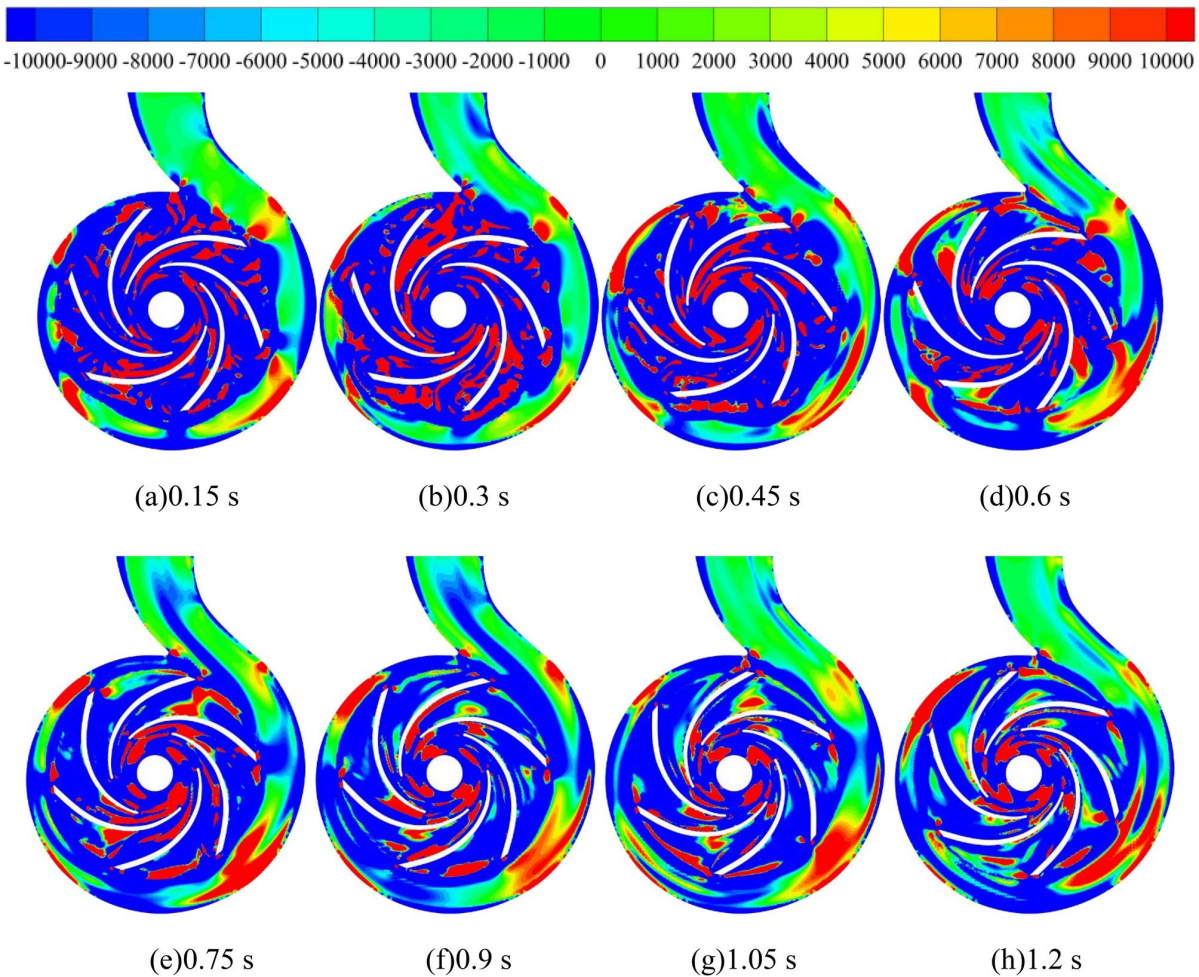
Vortex distribution in the middle section of the pump as turbine during medium-speed start-up (s^−2^).

Figure 19 shows the vortex distribution in the middle section of the pump as turbine based on the Q criterion. Before 0.3 s of the calculation process, the vortex in the impeller domain of the pump as turbine shows a speckle-like distribution, and the vortex values are very large, with values up to 10,000 s^−2^. In the volute domain, the vortex distribution is lumpy, with obvious transitions, and the vortex values in the partition tongue are larger. In addition, the vortex values near the volute II, IV, VI, and VIII sections are larger than those in the other parts. After 0.3 s of the calculation process, the vortex distribution area in the impeller domain starts to increase as the turbine starts to rotate the impeller, especially in the impeller outlet position. In the volute domain, the fluid motion near the tongue is very violent, which shows that the vortex value near the tongue is larger compared with its surrounding position, and its local maximum vortex value reaches 10,000 s^−2^. In addition, with the increase of pump as turbine impeller speed, the fluid motion near section VI becomes more and more violent, and the area with the larger value of vortex near section VI increases continuously, and its area range is expanded from only near section VI at the beginning of impeller start to all over the impeller. The range of the region is expanded from the initial impeller start only in the vicinity of section VI to between section V and section VII. After 0.9 s of the calculation process, although the acceleration of the pump as turbine impeller has ended, the vortex distribution remains the same as that during the acceleration period, showing the same distribution pattern. In summary, during the pump as turbine start-up process, the larger vortex distribution inside the pump as turbine is mainly concentrated near the impeller outlet and volute casing section V. There are also local vortex values near the spacer tongue and between the blades. In the impeller acceleration process, the vortex value of the volute casing section V grows larger.

**Figure 20:**
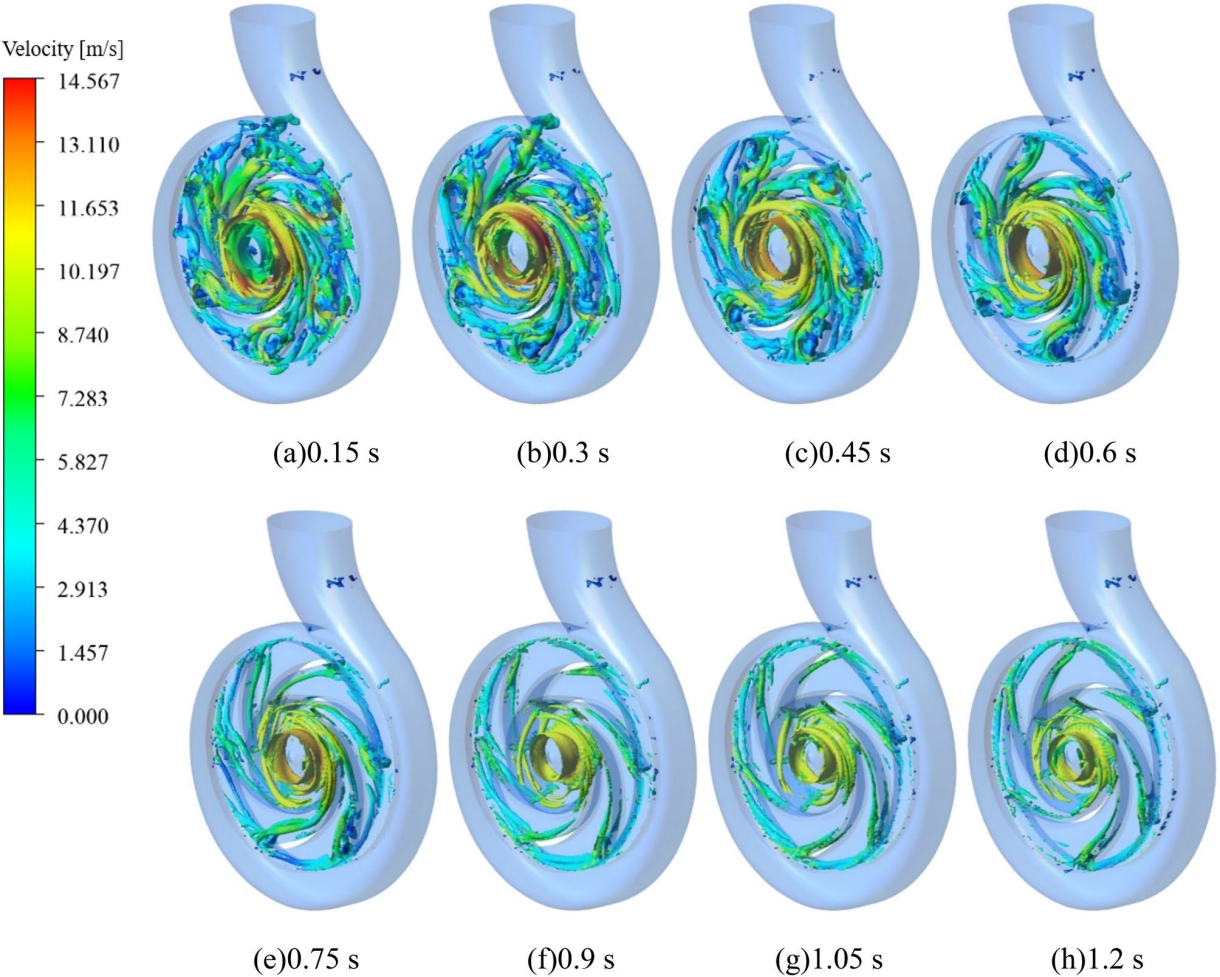
Evolution of the morphology of the vortex inside the pump as turbine during medium-speed start-up (m/s).

In addition, based on the Q criterion, the vortex evolution law of the pump as turbine during the medium speed start-up was identified by setting Q = 211,883 s^−2^ and the color of the vortex equivalent surface was expressed in terms of velocity, the results of which are shown in Fig. 20. It is obvious that the number of vortices in the impeller area is higher, especially at the impeller exit location, where both the speed and the number of vortices are significantly higher than in other locations, and the maximum local speed value at this location is 14.567 m/s. In the volute area, the vortices are mainly concentrated near the spacer tongue and the V section, and the number of vortices in the rest of the volute is very small, and the speed value of these vortices is also smaller, only 3 m/s. At the same time, the number of vortices in the domain decreases sharply during the pump as turbine start-up, and the number of vortices at 0.3 s is the highest in the whole start-up process, and then the number of vortices in the domain of both the impeller and the worm gear begins to decrease as the impeller of the pump as turbine accelerates to rotate. It is obvious to see that the vortex near the tongue gradually disappeared, and the number of a vortex between the blades also decreased, the reason for the above phenomenon may be that with the rotation of the pump as turbine impeller, the pump as turbine domain of fluid impact on the blades to reduce, and then lead to the reduction of its vortex number. In summary, in the process of system operation, the vortex in the pump as turbine domain is mainly distributed in the impeller domain, near the spacer tongue and the V section of the volute casing; with the accelerated operation of the pump as turbine impeller, the number of vortices in the whole domain decreases sharply, especially the vortex between the vanes decreases by an extremely large amount.

The entropy production theory is an irreversible process where the loss of mechanical energy is converted to internal energy, which is irreversible and eventually causes an increase in entropy production. According to the second thermodynamic theorem, there is also entropy production in the actual fluid system. Therefore, in order to more effectively explain the phenomenon of flow loss within the pump as turbine, this paper adopts the entropy production theory to explain the energy loss within the pump as turbine.

Usually, the flow within a centrifugal pump as turbine is a turbulent state, for which the entropy production^24^ has two parts: one part is caused by the time-averaged motion; the other part is caused by the velocity fluctuations in the transient state. Therefore, the entropy production rate $\dot{S}^{\prime \prime \prime }$ (EPR) can be expressed using the following equation.

19 $$\dot{S}^{\prime \prime \prime } = \dot{S}_{D}^{\prime \prime \prime } + \dot{S}_{D}^{\prime \prime \prime \prime }$$

The entropy production due to time-averaged and pulsation is as in Eqs. (20) and (21):

20 $$\dot{S}^{\prime \prime \prime }_{D} = \frac{2\mu }{T}\left[ {\left( {\frac{{\partial \overline{u}}}{\partial x}} \right)^{2} + \left( {\frac{{\partial \overline{v}}}{\partial y}} \right)^{2} + \left( {\frac{{\partial \overline{w}}}{\partial z}} \right)^{2} } \right] + \frac{\mu }{T}\left[ {\left( {\frac{{\partial \overline{v}}}{\partial x} + \frac{{\partial \overline{u}}}{\partial y}} \right)^{2} + \left( {\frac{{\partial \overline{w}}}{\partial x} + \frac{{\partial \overline{u}}}{\partial z}} \right)^{2} + \left( {\frac{{\partial \overline{v}}}{\partial z} + \frac{{\partial \overline{w}}}{\partial y}} \right)^{2} } \right] \,$$

21 $$\dot{S}_{{D^{\prime } }}^{\prime \prime } = \frac{{2\mu_{eff} }}{T}\left[ {\left( {\frac{{\partial u^{\prime } }}{\partial x}} \right)^{2} + \left( {\frac{{\partial v^{\prime } }}{\partial y}} \right)^{2} + \left( {\frac{{\partial w^{\prime } }}{\partial z}} \right)^{2} } \right] + \frac{{\mu_{eff} }}{T}\left[ {\left( {\frac{{\partial v^{\prime } }}{\partial x} + \frac{{\partial u^{\prime } }}{\partial y}} \right)^{2} + \left( {\frac{{\partial w^{\prime } }}{\partial x} + \frac{{\partial u^{\prime } }}{\partial z}} \right)^{2} + \left( {\frac{{\partial v^{\prime } }}{\partial z} + \frac{{\partial w^{\prime } }}{\partial y}} \right)^{2} } \right]$$

where $\dot{S}^{\prime \prime \prime }_{D}$ is the velocity average entropy yield; $\dot{S}^{\prime \prime \prime }_{D^{\prime}}$ is the velocity pulsation entropy yield; *μ* is the kinematic viscosity; $\overline{u}$, $\overline{v}$, $\overline{w}$ are the time-averaged velocities; $u^{\prime}$, $v^{\prime}$, $w^{\prime}$ are the pulsation velocities; *T* is the temperature, and the temperature is set as a constant 293 K in the calculation; $\mu_{eff}$ is the effective kinematic viscosity, as shown by Eq. (22):

22 $$\mu_{eff} = \mu + \mu_{t}$$

where $\mu_{t}$ is the turbulent motion viscosity.

$\dot{S}^{\prime \prime \prime }_{D}$ can be solved directly by numerical calculations, while $\dot{S}^{\prime \prime \prime }_{D^{\prime}}$ cannot be solved directly by numerical calculations. According to the local entropy production theory of Kock^24^, the entropy production due to velocity fluctuations is related to ε or ω of the turbulence model. Therefore, in the SST *k-ω* turbulence model^25^, the local entropy production due to velocity fluctuations is given in Eq. (23):

23 $$\dot{S}_{{D^{\prime \prime \prime } }} = \alpha \frac{\rho \omega k}{T}$$

where $\alpha$ = 0.09, $\omega$ is the turbulent vortex frequency, s^−1^;* k* is the turbulence intensity, m^2^/s^2^

However, due to the strong wall effect of entropy yield and the more pronounced time-averaged term, the entropy yield near the wall is calculated by the following:

24 $$S_{pro,W} = \int_{S} {\frac{{\vec{\tau } \cdot \vec{v}}}{T}} dS$$

where $\tau$ is the wall shear stress, Pa; *S* is the area, m^2^; $v$ is the near-wall velocity, m/s.

Therefore, the total entropy yield in the computational domain of the whole system is calculated as follows:

25 $$S_{{{\text{pro}}}} = \int_{V} {\dot{S}_{D}^{\prime \prime \prime } } dV + \int_{V} {\dot{S}_{{D^{\prime } }} }^{\prime \prime \prime } dV + \int_{S} {\frac{{\vec{\tau } \cdot \vec{v}}}{T}} dS$$

**Figure 21:**
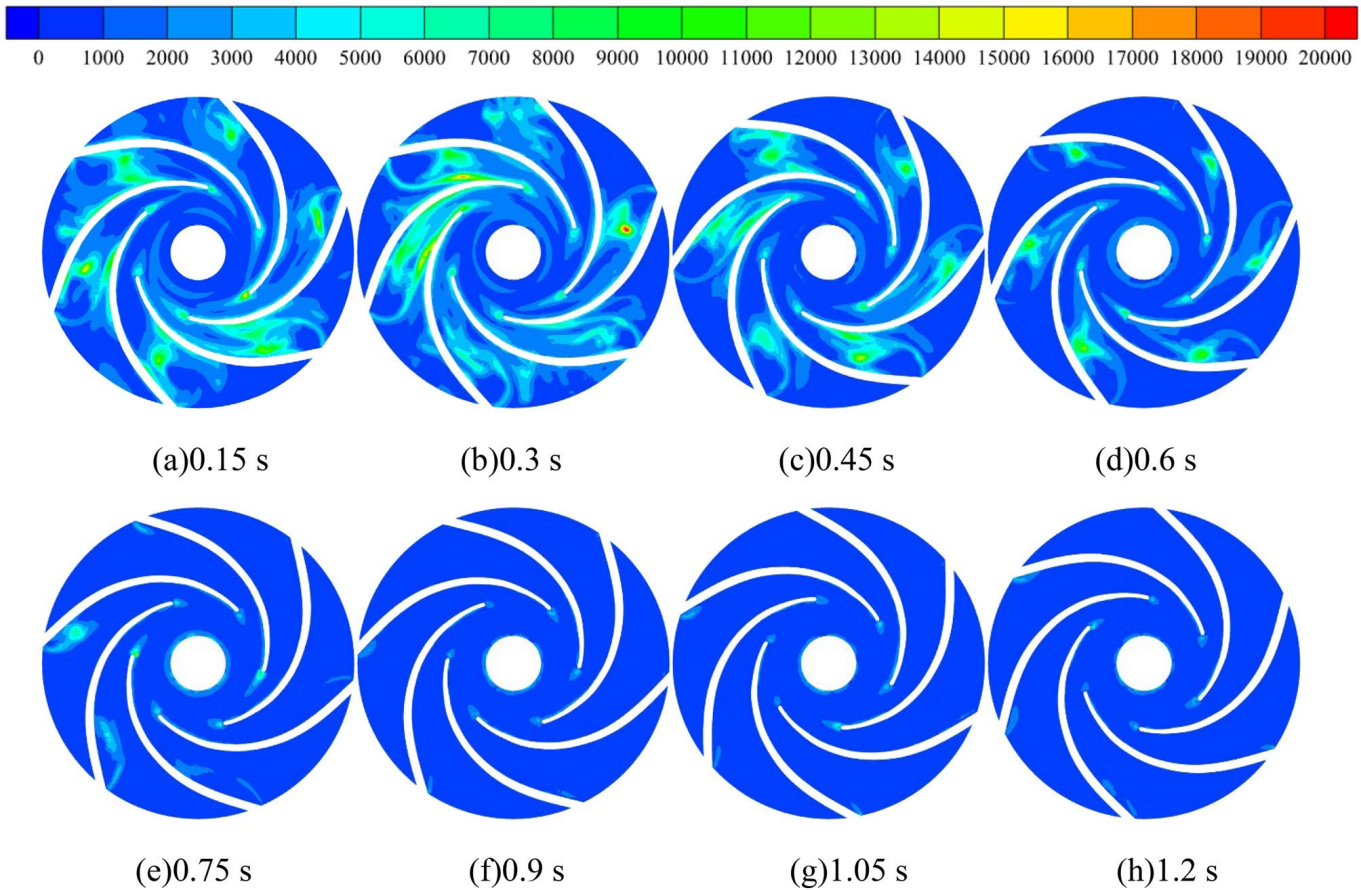
Entropy production distribution in the domain of the pump as turbine impeller during medium-speed start-up (W/(m^3^·K)).

Figure 21 shows the distribution of entropy production within the impeller domain of the pump as turbine. As can be seen from the figure, the loss within the impeller domain is mainly concentrated between the blades, and the loss at the impeller outlet is smaller. With the operation of the pump as turbine impeller, the entropy production value within the impeller domain also decreases, that is, the loss within the impeller domain decreases. In the calculation moment 0.3 s, the maximum entropy production distribution area in the impeller, that is, the maximum energy loss, the maximum entropy production value in its domain can reach 15,000 W/(m^3^·K). In the pump as turbine impeller acceleration process, the domain of the impeller within the entropy production distribution sharply reduced, in the calculation moment 0.9 s, in addition to the blade tip and blade pressure surface still exists entropy production, the rest of the position no entropy production distribution, and its value is also very small. After the uniform rotation of the impeller, the distribution of entropy production in the impeller is very small. This shows that the energy loss in the turbine impeller domain is reduced sharply during the acceleration of the pump as turbine impeller.

### Valves

**Figure 22:**
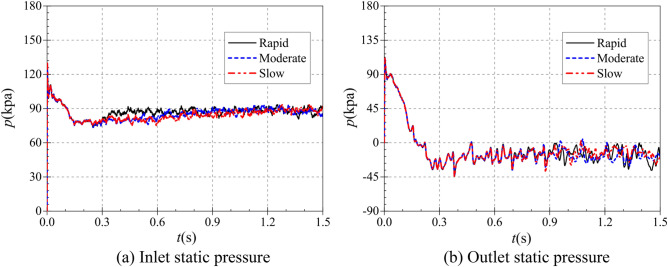
Valve inlet and outlet static pressure. (**a**) Inlet static pressure; (**b**) Outlet static pressure.

The inlet and outlet pressure evolution curves of the valve for three different start-up acceleration scenarios are shown in Fig. 22. Figure 22a shows the static pressure evolution at the valve inlet. Since the pressure at the valve inlet mainly depends on the outlet pressure of the pump as turbine, and the pump as turbine impeller does not start to rotate before 0.3 s of the calculation process and is in a completely stationary state, the static pressure curves under three starting accelerations are the same, all showing a rapid decline, then a rapid rise, and then a fluctuating downward evolutionary trend. Among them, the instantaneous static pressure value is 101.756 kPa when the inlet pressure drops rapidly from 134.081 kPa at 0.001 s of the calculation process to 0.011 s; after that, the inlet pressure increases rapidly and reaches a local maximum value of 111.083 kPa at 0.017 s of the calculation process, after which the inlet pressure starts to show a decrease again and reaches After that, the inlet pressure starts to decrease again and reaches 78.953 kPa at 0.3 s of the calculation process. After 0.3 s of the calculation process, the three curves started to rise in their respective forms as the pump as turbine impeller started to rotate with different accelerations. It can be found that despite the differences in the rising curves, they still have the same trend of slow growth to their respective stable values, and the growth all shows a fluctuating rise. In the rapid start-up process, the valve inlet pressure reaches a stable value of 88.262 kPa at the calculated time of about 0.43 s. After reaching the stable value, the valve inlet pressure starts to oscillate up and down within certain amplitude. During the medium-speed start-up, the valve inlet pressure reaches a stable value of 86.766 kPa at about 0.97 s. During the slow start-up, the valve inlet pressure reaches a stable value of 89.309 kPa at about 1.446 s.

Figure 22b shows the instantaneous pressure diagram of the valve outlet for different start-up scenarios. Unlike the inlet pressure, the valve outlet pressure curves are very similar in the three different starting conditions, which all show a fluctuating decline and start to fluctuate up and down periodically around 0.5 s at the time of calculation.

In summary, during the start-up process, the instantaneous inlet pressure curves under different start-up acceleration scenarios have the same rising trend, and the time required for the inlet static pressure to reach a stable value shows a certain lag relative to the pump as turbine speed rise time; the pump as turbine start-up acceleration has an extremely weak effect on the valve outlet pressure.

**Figure 23:**
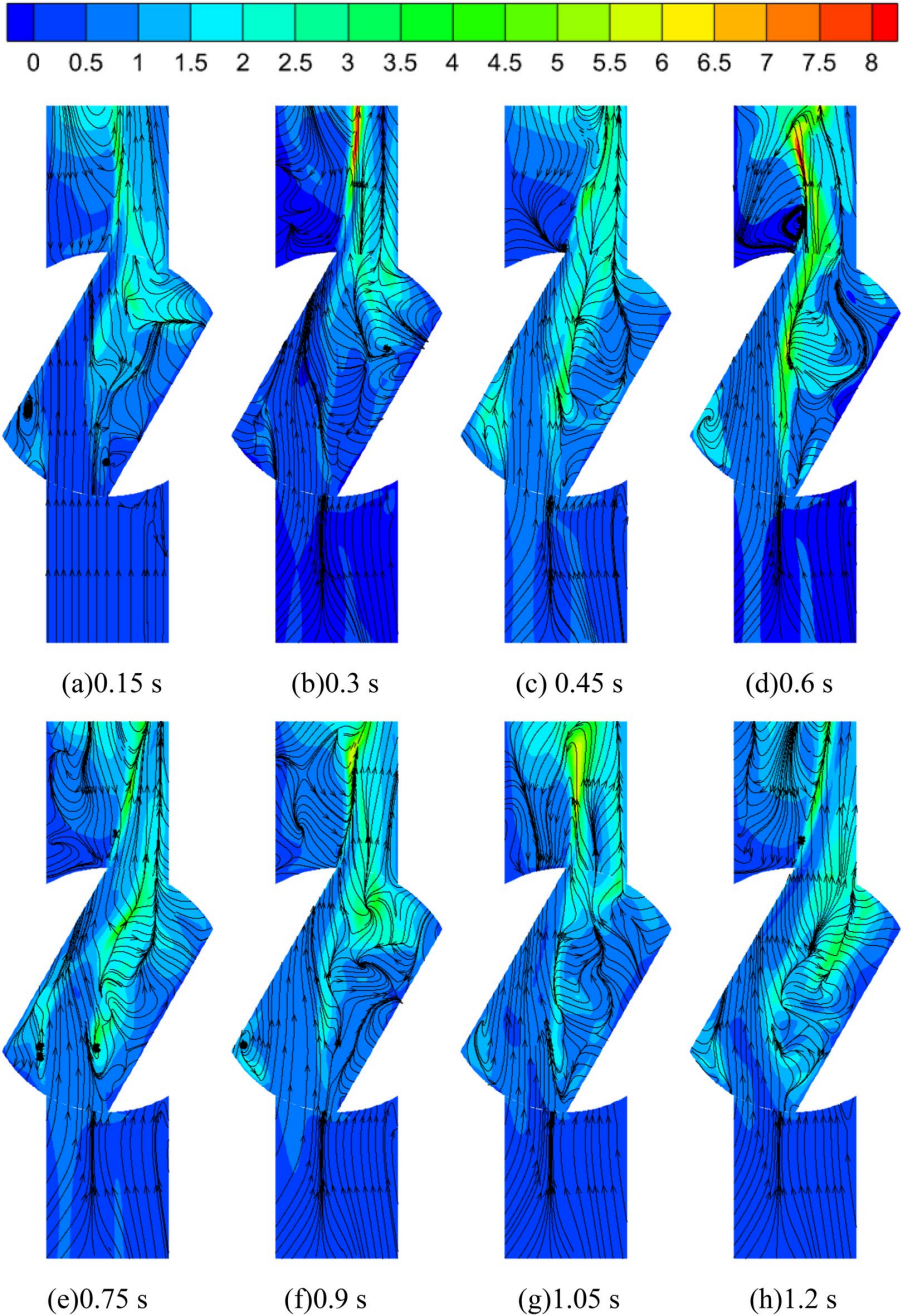
Turbulent kinetic energy distribution of the valve cross-section during medium-speed start-up (m^2^/s^2^).

The turbulent kinetic energy distribution and velocity flow line of the valve cross-section during the medium-speed start-up is shown in Fig. 23. On the whole, the turbulent kinetic energy in the valve cross-section is mainly concentrated in the middle and outlet section of the valve, and the turbulent kinetic energy distribution in the inlet section of the valve is very little; the velocity flow line in the inlet section of the valve is more uniform, and the velocity flow line distribution in the outlet section is very chaotic, and there is a vortex in the lower left position of the middle section of the valve. In addition, with the start of the pump as turbine, the turbulent kinetic energy in the valve domain showed a trend of first increasing and then decreasing. In the calculation moment 0.3 s, the turbulent kinetic energy distribution of the valve outlet section is larger, and its maximum turbulent kinetic energy value is 8 m^2^/s^2^, while the turbulent kinetic energy distribution of the valve inlet section and the middle section is less, and the energy loss is also less. The turbulent kinetic energy distribution in the valve domain at 0.6 s is the largest and the maximum turbulent kinetic energy reaches 8 m^2^/s^2^. At the same time, due to the large turbulent kinetic energy distribution, the flow line distribution at 0.6 s is also very confusing, and there is a large vortex at the junction of the middle section and the exit section, which causes more obvious energy loss. In the calculation moment 0.9 s, the pump as turbine impeller accelerated rotation ended and began to maintain uniform rotation, the turbulent kinetic energy distribution in the outlet section decreased, and the maximum turbulent kinetic energy value decreased to about 5 m^2^/s^2^.

In summary, in the pump as turbine system start-up process, the turbulent kinetic energy distribution in the valve flow field showed a trend of first increasing and then decreasing, the flow line distribution in the valve inlet section was more uniform, and the flow line distribution in the middle section and outlet section was very chaotic. The flow line distribution in the inlet section of the valve is more uniform, and the flow line distribution in the middle section and the outlet section is very chaotic, causing certain energy loss.

In the pump as turbine system, the valve, as an important component of the system, is more important for the study of its pressure loss under different start-up acceleration scenarios of the pump as turbine. Based on this, a dimensionless coefficient, the valve flow resistance coefficient, is introduced to better represent the pressure loss of the valve^26^, and its specific expression is shown in Eq. (26):

26 $$\xi = \frac{2\Delta p}{{\rho v^{2} }}$$

where Δ*p* is the valve pressure loss, kPa; *v* represents the valve inlet velocity, m/s.

**Figure 24:**
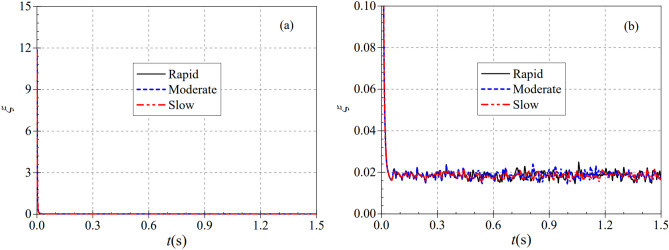
Transient flow resistance coefficients for different start-up conditions. (**a**) Overall diagram, (**b**) Partial diagram.

The instantaneous flow resistance coefficients under different start-up acceleration cases are shown in Fig. 24a,b. It can be seen that the curves of the valve flow resistance coefficients under different start-up acceleration cases have very similar trends, all of which are rapidly decreasing from a great value and showing a constant up and down cyclic fluctuation after reaching a relatively stable value. During the rapid, medium and slow start-up, the instantaneous flow resistance coefficient decreases rapidly from 11.858, 11.858 and 11.737, respectively, and reaches the minimum values of 0.165, 0.162 and 0.162 at 0.049, 0.053 and 0.056 s, respectively, at the time of calculation. fluctuates up and down, especially the pressure at the valve outlet position fluctuates up and down very significantly, thus causing the instantaneous flow resistance coefficient to fluctuate up and down in a certain range after reaching a relatively stable value. Under three different acceleration conditions, the average flow resistance coefficients after the impeller acceleration of the pump as turbine are 0.186, 0.188 and 0.184, respectively. it can be seen that the pump as turbine start-up speed has very little effect on the flow resistance coefficient of the valve.

**Figure 25:**
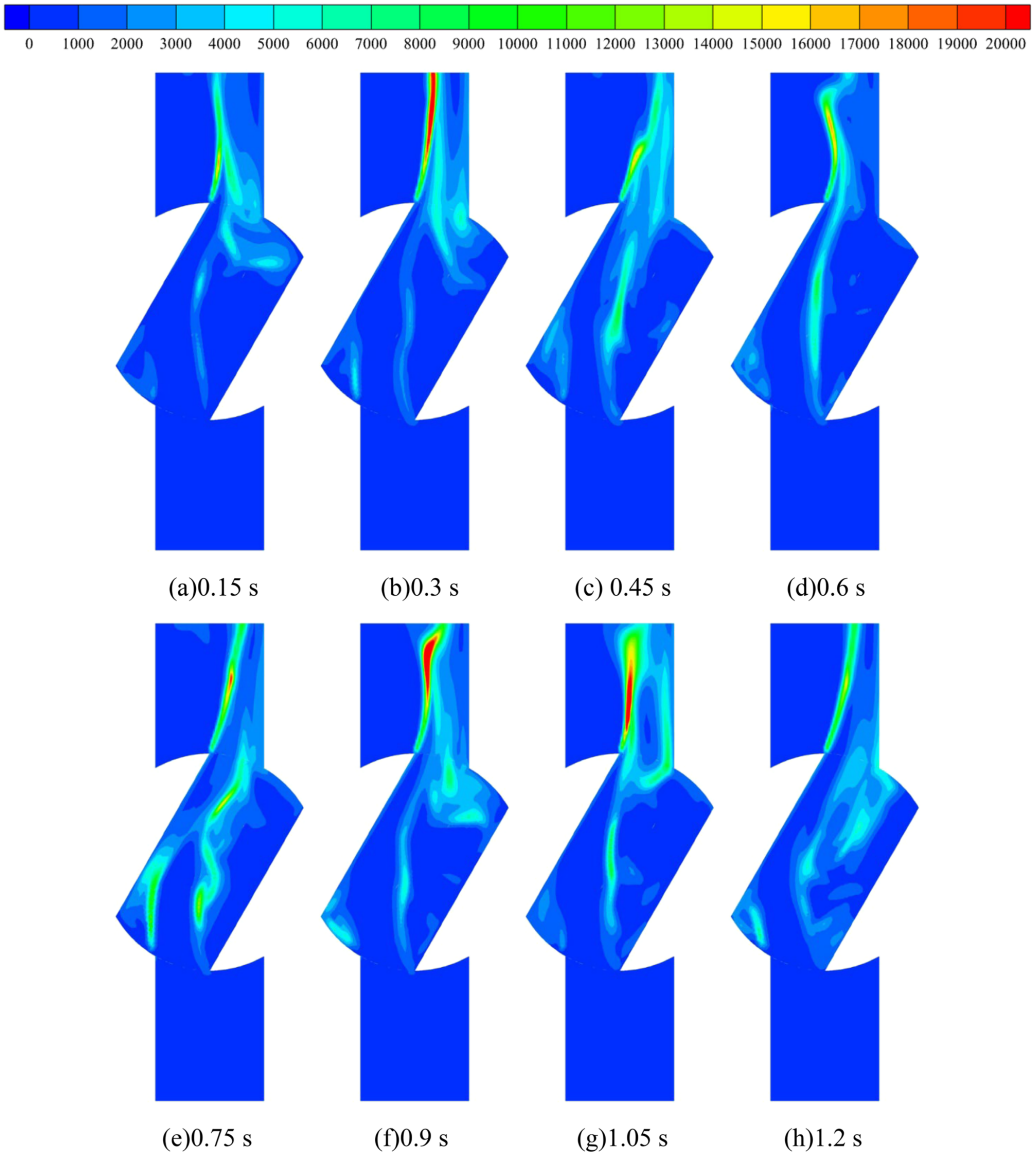
Entropy production distribution in the valve domain during medium-speed start-up (W/(m^3^·K)).

Figure 25 shows the entropy production distribution within the valve domain during the medium-speed start-up process. As can be seen from the figure, the entropy production in the valve domain is mainly concentrated in the outlet section of the valve, which has a high consistency with the turbulent kinetic energy distribution. At the calculation time of 0.3 s, the entropy production distribution of the valve outlet section shows a long strip, and its entropy production value is large, with the maximum value reaching 20,000 W/(m^3^·K). In the process of starting the pump as turbine impeller, the entropy production distribution in the valve domain shows a trend of first less and then increasing, and the entropy production distribution in the valve outlet section is the least at the calculation time 0.6 s and the most at the calculation time 0.9 s. In the process of starting the pump as turbine impeller, the entropy production distribution in the valve domain shows a trend of first less and then increasing. After the pump as turbine impeller is started, the entropy production distribution of the valve gradually decreases, i.e., the energy loss decreases. In summary, during the start-up of the pump as turbine system, the entropy production in the valve domain is mainly distributed in the valve outlet section, and its energy loss shows a trend of first decreasing and then increasing.

### Tank analysis

The tank is an important part of the pump as the turbine system, due to the introduction of the bulkhead in the middle part of the tank, thus making part of the internal flow field of the tank present more complex hydraulic characteristics, while part of the internal flow field of the tank presents a more stable flow state. Based on this, a series of hydraulic analyses of the internal flow field of the tank was carried out.

**Figure 26:**
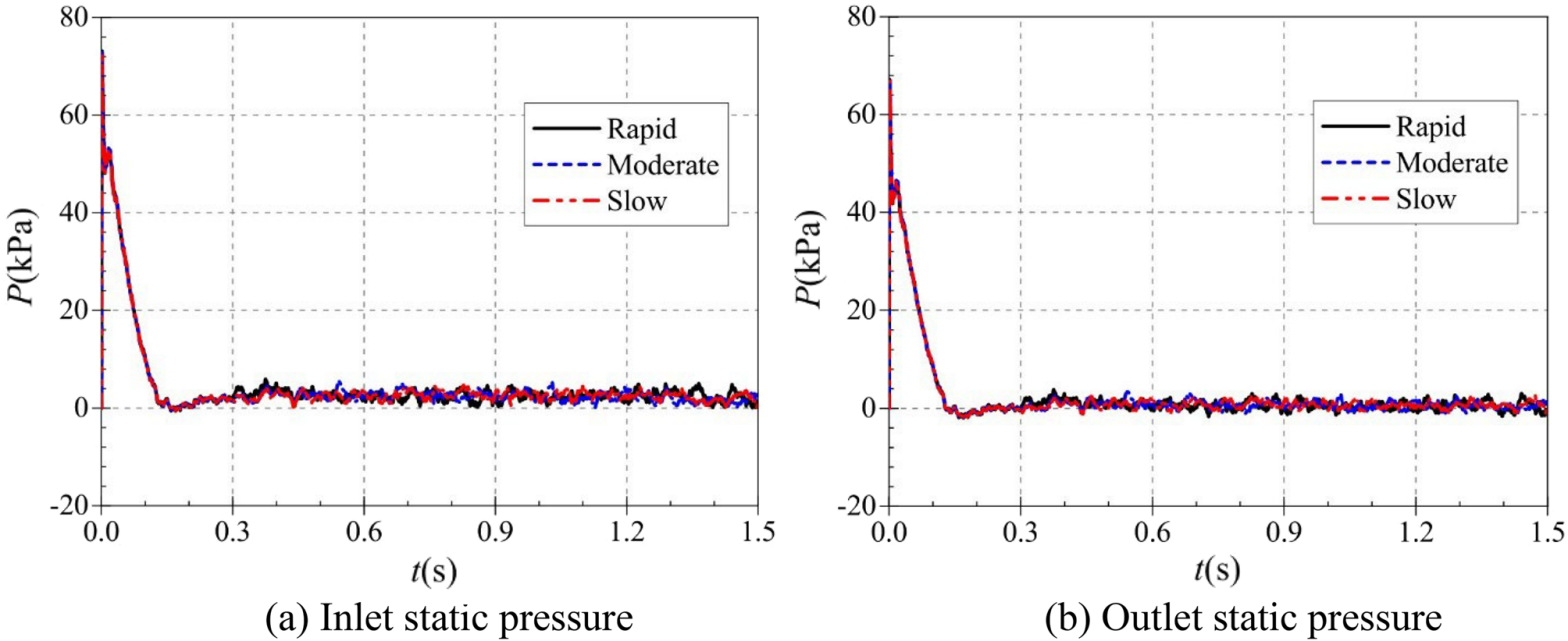
Instantaneous static pressure rise characteristics at different operating conditions. (**a**) Inlet static pressure, (**b**) Outlet static pressure.

Under different starting acceleration conditions, the static pressure rise characteristics of the tank inlet and outlet are shown in Fig. 26. Figure 26a,b show the instantaneous static pressure rise curves at the inlet and outlet of the tank, respectively. As the tank is far from the pump as turbine, therefore, the tank inlet and outlet pressure curves at different starting accelerations are very similar trends, are first rapid decline, then a small increase, then a rapid decline to the lowest point, and finally slowly rise to a stable value of the trend. For the tank inlet, the turbine impeller did not start to rotate under the three operating conditions until 0.3 s at the calculation moment, so the three curves completely overlapped, all starting from the highest value of 77.121 kPa at the beginning of the start and dropping rapidly, reaching a very small value of 47.336 kPa at 0.08 s at the calculation moment, and then rising to a local extreme value of 53.279 kPa at 0.02 s at the calculation moment. 53.279 kPa, then decreases rapidly again, reaching a minimum value of − 0.655 kPa at 0.17 s, after which the curve starts to fluctuate and rises until it ends at 0.3 s. After 0.3 s of the calculation moment, the three rising curves show some deviation because the pump as turbine impeller starts to run at different accelerations, but their overall trend remains the same, and the average pressure of the tank inlet after the pump as turbine impeller accelerates is 2.487 kPa, 2.137 kPa and 1.956 kPa respectively. For the tank outlet, it can be seen that the water tank The pressure change trend of the outlet is highly consistent with that of the tank inlet, both of which are the trend of rapid decrease, then small increase, then rapid decrease to the lowest point, and finally slow increase to the stable value. Therefore, the tank pressure loss is about 1.832 kPa, 1.565 kPa and 1.373 kPa for three acceleration conditions: rapid, medium and slow, respectively.

In summary, in different acceleration situations, the lower the acceleration of the pump as turbine impeller, the smaller the pressure loss of the water tank in the circulation system.

**Figure 27:**
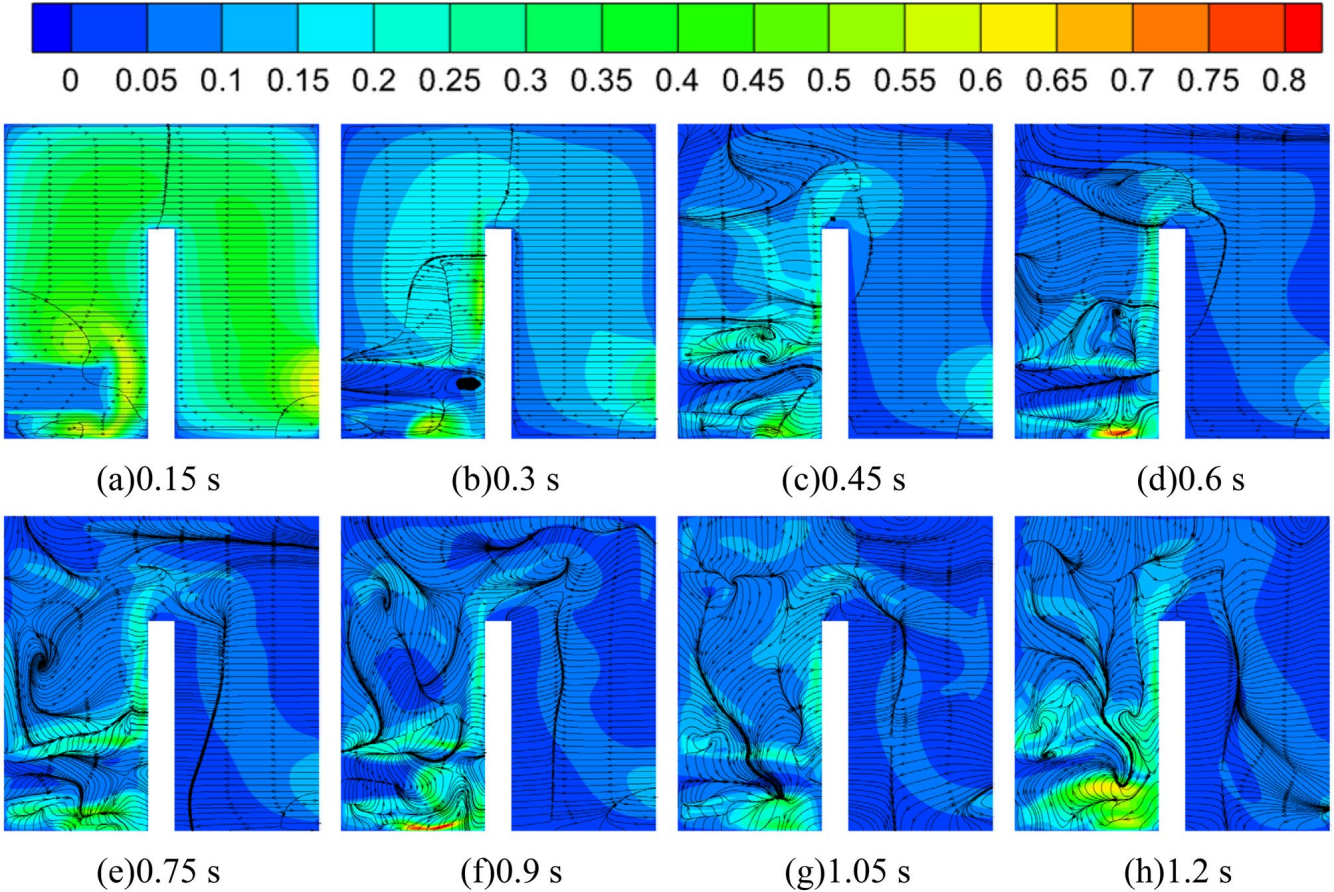
Turbulent kinetic energy and streamline the distribution of tank cross-section during medium-speed start-up (m^2^/s^2^).

Figure 27 shows the turbulent kinetic energy distribution and velocity flowline distribution in the tank mid-section during the medium-speed start-up. It can be found that the velocity flow line distribution on the inlet side of the tank is relatively regular, while the velocity flow line distribution on the outlet side of the tank is very complicated, especially since the flow line distribution near the outlet of the tank is very disordered. In addition, the streamlined distribution changes dramatically with the rotation of the pump as turbine impeller. At the calculation time of 0.15 s, as the pump as turbine impeller does not start to rotate, the streamlined distribution inside the whole tank is more regular at this time, and the turbulent kinetic energy at the inlet of the tank has a local extreme value area, the maximum value of which reaches 0.65 m^2^/s^2^, while the turbulent kinetic energy value at the outlet of the tank is relatively small, and its value is around 0.1 m^2^/s^2^. 0.1 m^2^/s^2^. At 0.3 s, the pump started to rotate as the turbine impeller, and the flow distribution on the left side of the tank baffle began to be disturbed, especially the fluid flow from the tank outlet to the baffle area was very violent, and a vortex appeared near the baffle position. Compared with the calculation moment 0.15 s, the velocity distribution inside the tank becomes relatively chaotic, and in the left domain of the tank, the closer to the baffle, the larger its velocity value. At the calculation moment of 0.45 s, as the impeller of the pump as turbine rotates faster, the fluid flow inside the tank becomes more violent, and the distribution of flow lines on the left side of the tank baffle is more turbulent compared with the calculation moment 0.15 s and 0.3 s. In addition, the flow lines on the upper side of the baffle become turbulent after 0.45 s. For the velocity, it is obvious that the turbulent kinetic energy value near the baffle and the tank outlet is larger, and its maximum value is around 0.55 m^2^/s^2^. At the calculation moment 0.6–0.9 s, the pump as turbine is in the process of accelerated rotation, when the flow in the domain of the tank is also very violent. It can be seen from the streamline distribution, except for the inlet side of the tank, the streamline distribution in other parts is very turbulent, especially in the area to the left of the baffle, with the accelerated rotation of the pump as turbine impeller, the number of its vortex is increasing, which may be due to the fluid flow in the pipeline after the pump as turbine becomes gradually complex.

For the turbulent kinetic energy distribution, it can be found that a local high turbulent kinetic energy area appears at the bottom of the tank on the left side, and its maximum value can reach 0.8 m^2^/s^2^. After the calculation moment of 0.9 s, the turbulent kinetic energy distribution in the tank is consistent with the previous one, and the turbulent kinetic energy value on the left side of the baffle is obviously larger than that on the right side. In summary, it can be seen that the tank domain in the pump as turbine accelerated start process, the internal flow of the tank is very complex, and the flow of the outlet side of the tank is more complex than the inlet side; the turbulent kinetic energy distribution is mainly concentrated in the inlet side of the tank, especially in the position of the baffle and the bottom of the tank, and there exist the local extreme value of the turbulent energy.

## Discussion

**Figure 28:**
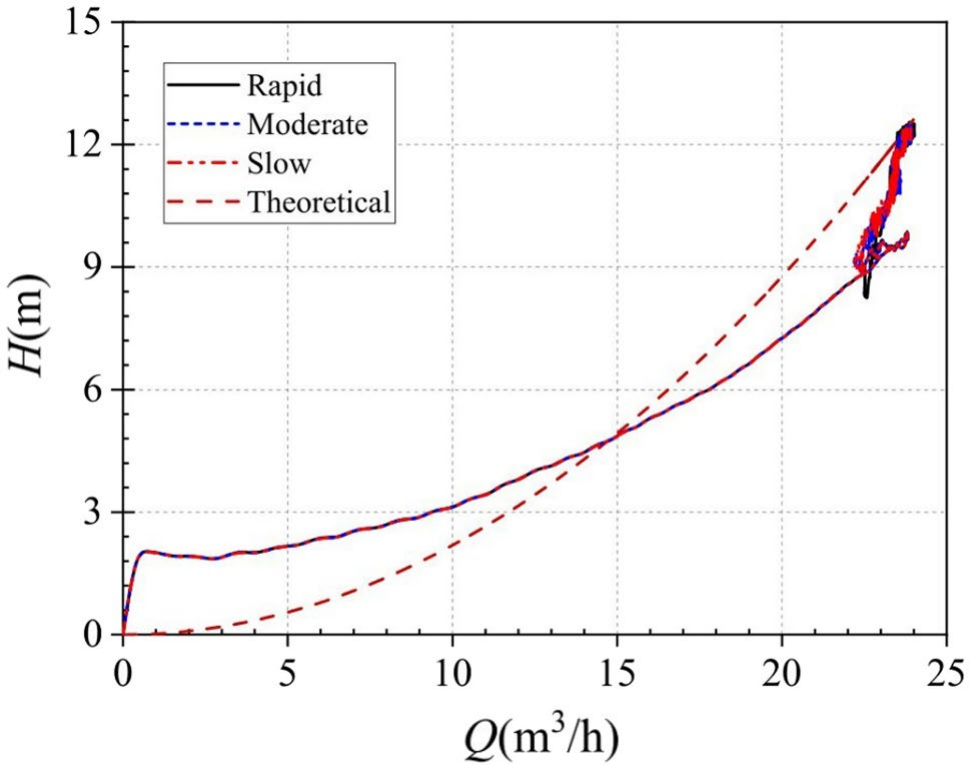
Instantaneous head-flow curve.

Similarity law is a very important law in the theory and design process of vane pumps. Based on this, this paper further analyzes the external characteristics of the pump as turbine start by plotting the theoretical head-flow curve and the instantaneous head-flow curve under different starting acceleration conditions, as shown in Fig. 28. In this simulation, the stable head and flow rate values after the start-up process is stabilized are known. According to the similarity law for centrifugal pumps proposed by Li and Zhang^27^, the theoretical head of the pump as turbine can be calculated using Eq. (27):

27 $$H = H_{0} \left( {\frac{Q}{{Q_{0} }}} \right)^{2}$$

where, *Q*_0_, *H*_0_ are the flow and head values after the end of the pump as turbine start respectively, *Q* is the actual flow rate at a moment of start-up, and H is the value of the pump as turbine head calculated by the law of similarity.

The instantaneous flow curves are identical up to 22.38 m^3^/h at three different start-up accelerations and follow a parabolic rule. After 22.38 m^3^/h, the instantaneous flow curve is very confusing due to the constant fluctuation of the flow rate in the system. By comparing with the theoretical curve, a large deviation was found between the two. This is because the theoretical value comes from the similarity law of the pump, which is essentially used to predict the stability performance. The initiation process studied in this paper is a typical non-stationary process. Therefore, the apparent difference between the two reflects the difference between stable and unstable operating conditions. This finding suggests that the similarity law for pumps does not apply to the prediction of turbine performance during start-up. Of course, more unsteady flow behavior inside pumps need to be deeply studied in the future works^28–30^.

## Conclusion

This paper focuses on the transient hydraulic and internal flow characteristics of the pump as turbine systems under three different start-up acceleration scenarios, focusing on the transient characteristics and energy losses of the pump as turbine, valve and tank overflow components during start-up, and the main conclusions obtained are as follows:

1. In the process of slow and medium speed start, the head curve and the speed curve grow similarly, both showing a similar linear upward trend; in the rapid start, the head curve shows a parabolic rise, and there is a sudden drop in the head at the beginning of the turbine start.
2. The larger vortex distribution inside the pump as the turbine is mainly concentrated at the impeller outlet and near the VI section of the volute, and there are also localized larger vortex values near the tongue and between the blades. In the process of impeller acceleration, the vortex value in the VI section of the volute increases more.
3. The entropy production in the domain of the pump as turbine impeller is mainly distributed between the blades, and the distribution is smaller at the impeller outlet; during the acceleration of the pump as turbine impeller, the entropy production distribution in the domain of the impeller decreases sharply.
4. The pump as turbine through the flow rate and outlet static pressure curve to reach a stable value of time, relative to the speed have hysteresis.
5. The instantaneous head-flow curve of the pump as the turbine is significantly different from the theoretical curve, which shows that the law of pump similarity does not apply to the performance prediction during the instantaneous start of the pump as turbine.
6. In the pump as turbine volute domain, for the same monitoring point, its pressure fluctuation amplitude with the pump as a turbine impeller start acceleration increases and decreases, and the pressure fluctuation amplitude is the largest at the slow start; for the same start acceleration situation, the pressure fluctuation amplitude of the monitoring point near the VIII section is the largest, and the fluctuation amplitude of the monitoring point near the volute is the smallest.
7. The pump as turbine inlet static pressure curve has a weak pressure shock phenomenon, while the flow curve has a flow shock phenomenon.
8. The turbulent kinetic energy in the tank domain is mainly concentrated on the inlet side of the tank; the middle baffle of the tank has a significant impact on the interior of the tank.
9. The entropy production in the valve domain is mainly distributed in the valve outlet section, the energy loss is first reduced and then increased, and the pump as turbine start acceleration on the valve flow resistance coefficient is very weak.
10. The turbulent kinetic energy distribution in the flow channel of the pump as turbine impeller gradually decreases; the turbulent kinetic energy distribution in the valve flow domain increases first and then decreases, and the flow line distribution in the middle and outlet section is disturbed.

## Data Availability

The data used to support the findings of this study are available from the corresponding author upon request.
